# Integrating the skin and blood transcriptomes and serum proteome in hidradenitis suppurativa reveals complement dysregulation and a plasma cell signature

**DOI:** 10.1371/journal.pone.0203672

**Published:** 2018-09-28

**Authors:** Lauren K. Hoffman, Lewis E. Tomalin, Gregory Schultz, Michael D. Howell, Niroshana Anandasabapathy, Afsaneh Alavi, Mayte Suárez-Fariñas, Michelle A. Lowes

**Affiliations:** 1 Albert Einstein College of Medicine, Bronx, New York, United States of America; 2 Department of Population Health and Science Policy, Department of Genetics and Genomics, Icahn School of Medicine at Mount Sinai, New York, New York, United States of America; 3 Department of Obstetrics and Gynecology, University of Florida, Miami, Florida, United States of America; 4 Incyte Corporation, Wilmington, Delaware, United States of America; 5 Department of Dermatology, Weill Cornell Medical College, New York, New York, United States of America; 6 Department of Medicine, Division of Dermatology, Women’s College Hospital, University of Toronto, Toronto, ON, Canada; 7 The Rockefeller University, New York, New York, United States of America; University of Naples, ITALY

## Abstract

Hidradenitis suppurativa (HS) is a chronic skin disease of the pilo-sebaceous apocrine unit characterized by significant inflammation and an impaired quality of life. The pathogenesis of HS remains unclear. To determine the HS skin and blood transcriptomes and HS blood proteome, patient data from previously published studies were analysed and integrated from a cohort of patients with moderate to severe HS (n = 17) compared to healthy volunteers (n = 10). The analysis utilized empirical Bayes methods to determine differentially expressed genes (DEGs) (fold change (FCH) >2.0 and false discovery rate (FDR) <0.05), and differentially expressed proteins (DEPs) (FCH>1.5, FDR<0.05). In the HS skin transcriptome (lesional skin compared to non-lesional skin), there was an abundance of immunoglobulins, antimicrobial peptides, and an interferon signature. Gene-sets related to Notch signalling and Interferon pathways were differentially activated in lesional compared to non-lesional skin. CIBERSORT analysis of the HS skin transcriptome revealed a significantly increased proportion of plasma cells in lesional skin. In the HS skin and blood transcriptomes and HS blood proteome, gene-sets related to the complement system changed significantly (FDR<0.05), with dysregulation of complement-specific DEGs and DEPs. These data point towards an exaggerated immune response in lesional skin that may be responding to commensal cutaneous bacterial presence and raise the possibility that this may be an important driver of HS disease progression.

## Introduction

The pathogenesis of the debilitating chronic skin disease hidradenitis suppurativa (HS) is still not well understood. A consistent observation is obstruction at the follicular infundibulum in apocrine gland bearing skin, and it seems possible that there are a number of initiating factors that could predispose to this development, reviewed in Hoffman et al. [1]. A proposed pathogenic pathway involves apocrine secretions accumulating in the hair follicle, which eventually ruptures onto the surface of the skin or into the dermis. Exposure to commensal bacteria then activates the innate immune system. Eventually the adaptive immune system is recruited. As this process is repeated, the inflammatory cascade leads to recurrent abscesses, suppurative dermal tunnels, and fibrosis. Important elements of the immune system that have been implicated include antimicrobial peptides (AMPs), Toll-like receptor 2 (TLR2), and cytokines such as IL-1, IL-6, IL-17, IL-12/23, and TNF. Many of the discoveries regarding HS to date have not been distinctive of HS, but reflect general cutaneous inflammatory responses to insults [2], along with a lack of resolution of the inflammatory process [3].

Even though a number of studies have contributed to defining the inflammatory milieu of HS by identifying potential individual biomarkers, only two publications describe the HS skin transcriptome [4, 5], and one evaluated the proteome in HS patients [6]. The skin microarray data by Blok et al. has previously been re-analyzed to show alteration of sphingolipid metabolism pathways in HS [7], as well as identification of novel therapeutics for HS [8]. However, mining from these powerful “hypothesis-free” omics platforms can still reveal novel pathways to evaluate.

In an effort to better understand HS disease progression and pathogenesis, we utilized a systems biology approach to integrate this omics data from a single cohort of HS patients presented in the two Blok et al. studies [4, 6] and a variety of bioinformatics techniques were used to mine the integrated data. In the HS skin transcriptome, we found abundant immunoglobulin and antimicrobial peptide genes, an interferon signature, and plasma cells were identified. The gene-set of the complement pathway was differentially activated across the HS skin and blood transcriptomes and the HS blood proteome. Furthermore, in the HS serum proteome, dysregulation of individual complement components was evident. These data suggest that an exuberant immune response may be driving HS chronicity. Although the factors responsible for this aberrant immune response are not yet elucidated, it is possible that dysbiotic cutaneous commensal bacteria may be playing a driving role in HS disease progression.

## Materials and methods

### Overview of data

Raw microarray and proteomic profiles were downloaded from Gene Expression Omnibus (GEO, GSE79150) using GEOQuery or raw proteomic data was obtained from the investigators of published studies [4–6, 9]. A summary of these studies, including number of patients, samples, lesions that were biopsied, and omic platform are in S1 Table. The data for analysis was from 17 patients with moderate-to severe HS, who had matching clinical data, skin and blood transcriptome and serum proteome (S1 File Page A) [4, 6]. An overview of patient demographic information can be found in S2 Table. There were two different groups of healthy volunteers for skin biopsies and blood. No demographic information is available for these healthy volunteers. Additional proteomic data were from patients with moderate-to-severe atopic dermatitis (AD), psoriasis (PS) and contact dermatitis (CD) [9].

### Statistical analysis

Statistical analysis was carried out using R-language (R-project.org) and packages available through the Bioconductor project (www.bioconductor.org). The study sample size was based on the design of the original studies. A homogeneous analytical pipeline was utilized.

Differences in profiles of gene and protein expression were assessed using linear mixed-effect models with tissue-type as a fixed factor, and patient as a random effect. Blood gene/protein expression profiles were compared between HS and healthy controls using linear models with disease-state. Models were fit in the *limma* package framework and hypothesis of interest were tested via contrasts using the moderated paired t-test [10]. P-values were corrected for multiple hypothesis testing and are presented with FDR values (significance level <0.05).

Gene Set Variation Analysis (GSVA) is a pathway-based analysis approach that provides an overall pathway or gene-set activity score for each sample [11]. GSVA z-scores for Hallmark gene-sets from the Molecular Signature Database (MSigDB) (http://software.broadinstitute.org/gsea/msigdb) and other gene-sets were calculated from skin and blood transcriptome profiles using the GSVA package and compared across conditions using the same linear and mixed-effect models as the expression data. When calculating scores for proteomic data, gene identifiers of each gene name were converted to protein names prior to performing GSVA.

CIBERSORT allows an estimation of the abundance of immune cells for each sample using tissue gene expression profiles [12]. Levels of skin and blood immune cells were estimated from transcriptomic profiles using the CIBERSORT web app (https://cibersort.stanford.edu/index.php) and then analysed using the same analytic framework as described above. When comparing proteomic profiles from different studies (HS vs AD, PS and CD), batch adjustment was required, performed with the normal samples present in both studies using the R function *ComBat* from the *sva* package. PVCA analysis was used to assess that batch effect was correctly eliminated.

## Results

### HS skin transcriptome was dominated by immunoglobulins, antimicrobial peptides (AMPs), an interferon signature, and plasma cells

An overview of our integrative analysis is shown in S1 File Page A, and demographic features of the HS patients are presented in S2 Table. The microarray gene expression profiles from HS lesional (LS) skin to matched HS non-lesional (NL) skin were modelled to identify differentially expressed genes (DEGs) (FCH>2.0, FDR<0.05). The list of DEGs was refined to filter transcripts representing the same genes, and 422 (643 probes) unique upregulated DEGs and 310 (430 probes) downregulated DEGs were identified (FCH for all unique genes in S4 Table). This signature was similar to the prior published transcriptome [4].

The top 50 unique DEGs with the largest expression change between LS versus NL in the HS skin transcriptome are listed in Table 1. Top upregulated DEGs in HS included immunoglobulin genes, AMPs (S100A7, A8, A9 and DEFB4), SERPINB3 and B4, CCL18, CXCL1, desmocollin 2 and fibrillin. Nine of the top 50 upregulated DEGs in HS were genes known to be upregulated in psoriasis (PS), including AMPs S100A7, A9, DEFB4, as well as SERPINB3 AND B4 [13]. Top downregulated DEGs in HS included sweat gland proteins, prolactin induced protein and dermcidin.

**Table 1:** Hidradenitis suppurativa skin transcriptome.

**Gene Name**	**Symbol**	**logFCH** **LSvsNL**	**FCH** **LSvsNL**	**Pval** **LSvsNL**	**FDR** **LSvsNL**
S100 calcium binding protein A7A	S100A7A	4.85	28.9	4.40E-08	8.78E-06
immunoglobulin heavy constant gamma 3	IGHG3	4.35	20.43	1.07E-07	1.51E-05
serpin family B member 4	SERPINB4	4.27	19.35	3.49E-06	0.000143
defensin beta 4A | defensin beta 4B	DEFB4A	3.8	13.89	6.80E-07	4.77E-05
S100 calcium binding protein A9	S100A9	3.53	11.54	7.68E-09	3.10E-06
ADAM like decysin 1	ADAMDEC1	3.49	11.22	8.25E-08	1.29E-05
immunoglobulin kappa constant	IGKC	3.23	9.41	8.66E-06	0.000271
tryptophan 2,3-dioxygenase	TDO2	3.13	8.75	2.13E-06	0.000102
transcobalamin 1	TCN1	3.12	8.67	2.24E-05	0.000528
aldo-keto reductase family 1 member B10	AKR1B10	3.07	8.38	8.76E-10	9.27E-07
serpin family B member 3	SERPINB3	3.01	8.03	2.51E-08	6.12E-06
S100 calcium binding protein A8	S100A8	2.97	7.83	8.27E-10	9.27E-07
C-C motif chemokine ligand 18	CCL18	2.95	7.72	4.62E-07	3.74E-05
immunoglobulin lambda variable cluster	IGLV	2.9	7.46	0.000123	0.00173
C-X-C motif chemokine ligand 1	CXCL1	2.79	6.93	1.56E-06	8.28E-05
immunoglobulin kappa variable 1D-13	IGKV1D-13	2.77	6.82	3.65E-05	0.000732
matrix metallopeptidase 12	MMP12	2.72	6.58	5.40E-05	0.000964
peptidase inhibitor 3	PI3	2.66	6.31	7.23E-07	4.99E-05
desmocollin 2	DSC2	2.65	6.28	2.44E-06	0.000113
SLAM family member 7	SLAMF7	2.6	6.05	3.25E-07	3.03E-05
ADAM metallopeptidase domain 12	ADAM12	2.58	6	5.69E-06	0.000201
fibrillin 2	FBN2	2.58	5.99	2.25E-08	5.67E-06
immunoglobulin heavy constant delta	IGHD	2.58	5.98	4.84E-05	0.000895
keratin 16	KRT16	2.57	5.93	5.38E-07	4.10E-05
apelin receptor early endogenous ligand	APELA	2.54	5.83	3.41E-10	6.99E-07
prolactin induced protein	PIP	-5.36	-41.21	8.98E-11	3.12E-07
dermcidin	DCD	-5.22	-37.25	2.55E-07	2.55E-05
secretoglobin family 2A member 2	SCGB2A2	-4.88	-29.38	6.71E-07	4.72E-05
adiponectin, C1Q and collagen domain containing	ADIPOQ	-4.5	-22.63	2.38E-11	1.76E-07
thyroid hormone responsive	THRSP	-3.82	-14.09	4.21E-05	0.000815
tetraspanin 8	TSPAN8	-3.78	-13.76	1.03E-09	9.98E-07
secretoglobin family 1D member 2	SCGB1D2	-3.76	-13.52	7.20E-08	1.20E-05
WNT inhibitory factor 1	WIF1	-3.71	-13.07	3.04E-11	1.76E-07
perilipin 1	PLIN1	-3.43	-10.78	2.05E-12	2.67E-08
3-hydroxy-3-methylglutaryl-CoA synthase 2	HMGCS2	-3.05	-8.27	1.43E-10	4.08E-07
betacellulin	BTC	-3.04	-8.21	3.65E-07	3.25E-05
leucine rich repeat containing G protein-coupled receptor 5	LGR5	-3.02	-8.1	2.63E-09	1.72E-06
complement C7	C7	-2.79	-6.91	6.85E-07	4.79E-05
fatty acid binding protein 7	FABP7	-2.75	-6.73	2.45E-05	0.000562
fatty acid binding protein 4	FABP4	-2.61	-6.09	3.81E-08	7.86E-06
Sp8 transcription factor	SP8	-2.56	-5.89	8.39E-08	1.29E-05
ATPase H+ transporting V0 subunit a4	ATP6V0A4	-2.52	-5.75	6.39E-05	0.00109
hypoxia inducible factor 3 alpha subunit	HIF3A	-2.43	-5.37	1.20E-08	3.99E-06
claudin 8	CLDN8	-2.41	-5.31	4.17E-05	0.000809
erb-b2 receptor tyrosine kinase 4	ERBB4	-2.4	-5.27	1.20E-06	6.95E-05
semaphorin 3E	SEMA3E	-2.4	-5.26	6.83E-08	1.18E-05
suppressor APC domain containing 1	SAPCD1	-2.35	-5.11	6.58E-13	1.14E-08
fibroblast growth factor binding protein 2	FGFBP2	-2.29	-4.9	5.60E-08	1.05E-05
keratin 77	KRT77	-2.29	-4.88	2.99E-05	0.000644
carbonic anhydrase 6	CA6	-2.28	-4.86	2.23E-05	0.000525

To further understand the HS skin transcriptome, GSVA scores were compared between LS and NL skin to identify differential activity of Hallmark and other published gene-sets (FDR<0.05; S1 File Page B). Gene-sets related to Notch signalling, Interferon alpha response, and Interferon gamma response showed the biggest changes in activity in LS versus NL skin. Gene-sets related to Complement and IL-6 JAK STAT3 signalling also showed a significant activation in LS skin.

GSVA scores were generated for up-regulated gene-sets in the following conditions, chosen for their potential relationship to pathogenesis (S1 File Page C): macrophage interferon signalling [14, 15]; IL-17 and TNF signalling in keratinocytes [16]; AD [17]; and PS [13]. All showed increased scores in HS skin. Nicastrin knockdown in keratinocytes induces type I interferon genes, but this gene-set was not differentially activated in the HS skin transcriptome (S1 File Page C).

**Fig 1:**
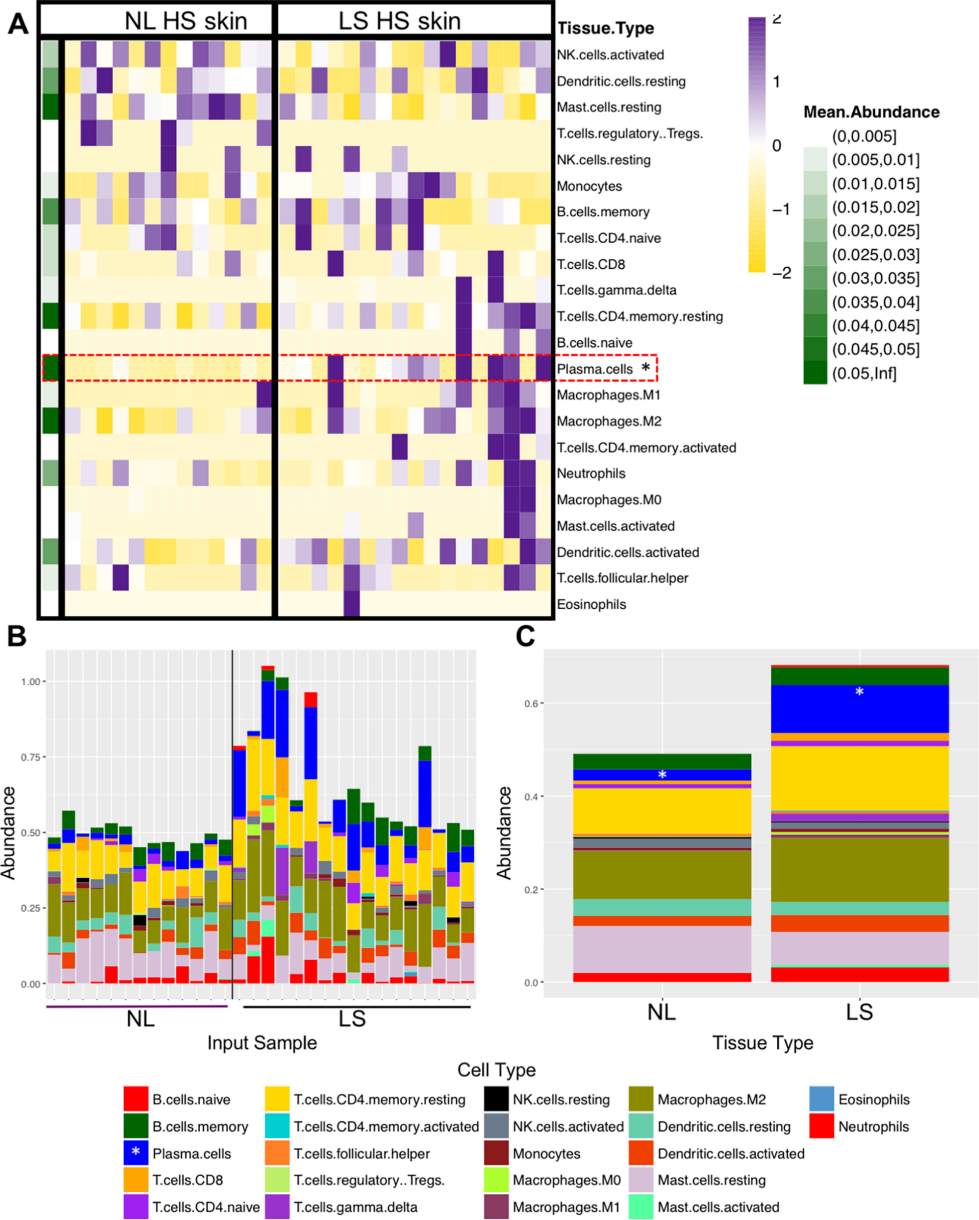
CIBERSORT analysis of HS skin transcriptome. (A) Heatmap showing normalized absolute abundance for each cell type in individual samples, mean abundance for each cell type is also displayed (left, green); (B) bar graph showing absolute abundance of each cell type in non-lesional (NL) and lesional (LS) samples; and (C) mean abundance of each cell type in all NL samples (left) and LS samples (right). Data from S4 Table. The abundance of plasma cells was significantly higher in LS versus NL skin (*FDR <0.05).

The analysis of immune cell abundance estimated using CIBERSORT identified only plasma cells as more abundant in LS than NL skin (FDR<0.05) (Fig 1).

### Minimal differences were observed in the HS blood transcriptome

The HS blood transcriptome was determined by comparing microarray gene expression of HS blood to that of healthy volunteers (S5 Table). There were no DEGs (FCH>1.5, FDR<0.05), which was similar to the observations of the prior investigators [4, 5]. However, despite null findings using the gene-based approach, the pathway-based approach identified global differential activation (S1 File Page D). Androgen response, protein secretion, and MTORC1 signalling were the top three differentially activated gene-sets, with Notch signalling and Complement gene-sets also showing increased scores in HS patients. This is described further in S1 Text. S1 File Page E presents the CIBERSORT analysis of the blood transcriptome.

### HS blood proteome

The HS blood proteome was determined by comparing expression of 1129 proteins in HS patients to that of healthy volunteers (SOMAscan platform). The prior analysis detected 54 dysregulated proteins, perhaps due to different modelling tools [6]. In our new analysis, there were 28 upregulated DEPs and 34 downregulated DEPs (FCH>1.5, FDR<0.05) (FCH for all proteins in S6 Table, DEPs shown in Table 2 and S1 File Page F). GSVA identified four differentially activated gene/protein-sets in this proteome (FDR<0.05), namely MYC targets VI, Reactive oxygen species pathway, Adipogenesis, and Complement (S1 File Page G). There was no overlap between the HS transcriptome DEGs and HS serum proteome DEPs, although the Complement pathway was activated in all three analyses. The unique HS proteomic signature is described further in S1 Text and S1 File Page H. The lack of response of the HS blood proteome to ustekinumab treatment is described in S1 Text.

**Table 2:** Hidradenitis suppurativa blood proteome.

**No.**	**Protein Name**	**Symbol**	**logFCH** **HSvsN**	**FCH** **HSvsN**	**Pval** **HSvsN**	**FDR** **HSvsN**
1	Glyceraldehyde-3-phosphate dehydrogenase	GAPDH, liver/GAPDH	3.62	12.32	4.68E-12	1.87E-09
2	Pyruvate kinase PKM	M2-PK/PKM2	2.72	6.58	4.96E-12	1.87E-09
3	GTP-binding nuclear protein Ran	RAN/RAN	1.81	3.51	1.55E-06	0.000118
4	Pulmonary surfactant-associated protein D	SP-D/SFTPD	1.63	3.09	6.33E-05	0.00196
5	Aflatoxin B1 aldehyde reductase member 2	Aflatoxin B1 aldehyde reductase/AKR7A2	1.62	3.08	1.18E-06	0.000102
6	Adenylate kinase isoenzyme 1	Myokinase, human/AK1	1.36	2.56	0.000357	0.00683
7	beta-adrenergic receptor kinase 1	BARK1/ADRBK1	1.32	2.49	1.90E-06	0.000126
8	Importin subunit beta-1	IMB1/KPNB1	1.25	2.37	9.40E-05	0.00259
9	C5a anaphylatoxin	C5a/C5	1.22	2.34	6.46E-06	0.000346
10	Alpha-soluble NSF attachment protein	SNAA/NAPA	1.19	2.29	1.31E-05	0.000592
11	Heterogeneous nuclear ribonucleoprotein A/B	hnRNP A/B/HNRNPAB	1.15	2.23	4.94E-07	6.20E-05
12	Proliferation-associated protein 2G4	PA2G4/PA2G4	1.03	2.04	1.74E-06	0.000123
13	Heterogeneous nuclear ribonucleoproteins A2/B1	hnRNP A2/B1/HNRNPA2B1	1.02	2.03	0.000311	0.0065
14	Ras-related C3 botulinum toxin substrate 1	RAC1/RAC1	0.94	1.92	1.41E-05	0.000611
15	Carbonic anhydrase 1	Carbonic anhydrase I/CA1	0.94	1.92	0.00124	0.0169
16	Adenylosuccinate lyase	PUR8/ADSL	0.87	1.83	0.00459	0.0432
17	N-acetyl-D-glucosamine kinase	NAGK/NAGK	0.86	1.81	1.93E-05	0.000808
18	Casein kinase II 2-alpha:2-beta heterotetramer	CK2-A1:B/CSNK2A1 CSNK2B	0.8	1.74	0.0028	0.0296
19	Mitogen-activated protein kinase 3	ERK-1/MAPK3	0.77	1.71	2.95E-05	0.00111
20	Catalase	Catalase/CAT	0.77	1.71	0.00326	0.0332
21	Rab GDP dissociation inhibitor beta	Rab GDP dissociation inhibitor beta/GDI2	0.76	1.69	6.53E-05	0.00196
22	Glycogen synthase kinase-3 alpha/beta	GSK-3 alpha/beta/GSK3A GSK3B	0.73	1.66	6.48E-06	0.000346
23	Ectonucleoside triphosphate diphosphohydrolase 5	ENTP5/ENTPD5	0.65	1.57	2.57E-06	0.000152
24	Stress-induced-phosphoprotein 1	Stress-induced-phosphoprotein 1/STIP1	0.64	1.55	0.00108	0.0152
25	X-ray repair cross-complementing protein 6	Ku70/XRCC6	0.62	1.54	0.00433	0.0411
26	Extracellular matrix protein 1	ECM1/ECM1	0.62	1.53	0.00246	0.027
27	Prostaglandin G/H synthase 2	COX-2/PTGS2	0.59	1.51	7.99E-07	8.65E-05
28	Proprotein convertase subtilisin/kexin type 7	PCSK7/PCSK7	0.59	1.51	0.000212	0.00487
1	Leukotriene A-4 hydrolase	LKHA4/LTA4H	-3.06	-8.37	2.28E-10	6.45E-08
2	Complement C3b	C3b/C3	-2.83	-7.09	4.82E-08	9.07E-06
3	Follicle stimulating hormone	FSH/CGA FSHB	-1.99	-3.98	0.000343	0.00667
4	Dual specificity protein phosphatase 3	DUS3/DUSP3	-1.78	-3.43	1.10E-08	2.49E-06
5	Complement C4b	C4b/C4A C4B	-1.64	-3.11	4.15E-05	0.00142
6	Carbonic anhydrase 13	Carbonic anhydrase XIII/CA13	-1.62	-3.08	9.47E-07	8.91E-05
7	Complement C3b, inactivated	iC3b/C3	-1.48	-2.79	7.66E-13	8.65E-10
8	Human Chorionic Gonadotropin	HCG/CGA CGB	-1.48	-2.78	0.00238	0.0269
9	Luteinizing hormone	Luteinizing hormone/CGA LHB	-1.41	-2.65	0.00198	0.0231
10	cAMP-dependent protein kinase catalytic subunit alpha	PRKACA/PRKACA	-1.36	-2.57	8.43E-07	8.65E-05
11	Tyrosine-protein phosphatase non-receptor type 11	SHP-2/PTPN11	-1.28	-2.42	8.32E-08	1.17E-05
12	Tyrosine-protein kinase Fyn	FYN/FYN	-1.1	-2.14	9.64E-05	0.00259
13	Platelet glycoprotein VI	GPVI/GP6	-1.05	-2.07	6.75E-06	0.000346
14	Inducible T-cell costimulator	ICOS/ICOS	-1.01	-2.01	6.58E-05	0.00196
15	Heat shock 70 kDa protein 1A/1B	HSP 70/HSPA1A	-0.98	-1.98	7.16E-08	1.16E-05
16	NSFL1 cofactor p47	NSF1C/NSFL1C	-0.99	-1.98	0.001	0.0143
17	Tyrosine-protein kinase BTK	BTK/BTK	-0.92	-1.89	0.000328	0.00662
18	C-C motif chemokine 28	CCL28/CCL28	-0.9	-1.87	0.00404	0.039
19	Tyrosine-protein kinase Fer	FER/FER	-0.87	-1.82	0.00109	0.0152
20	Midkine	Midkine/MDK	-0.8	-1.75	0.000757	0.012
21	Eukaryotic translation initiation factor 4 gamma 2	IF4G2/EIF4G2	-0.79	-1.73	0.000786	0.0122
22	Xaa-Pro aminopeptidase 1	XPNPEP1/XPNPEP1	-0.76	-1.7	0.000104	0.00273
23	Eukaryotic translation initiation factor 5	eIF-5/EIF5	-0.77	-1.7	0.000257	0.00557
24	Dual specificity mitogen-activated protein kinase kinase 2	MP2K2/MAP2K2	-0.75	-1.68	0.00013	0.00334
25	Myoglobin	Myoglobin/MB	-0.71	-1.64	1.24E-05	0.000584
26	Growth/differentiation factor 2	GDF2/GDF2	-0.68	-1.61	9.60E-06	0.000471
27	Complement C3	C3/C3	-0.66	-1.59	0.000443	0.00782
28	Translationally-controlled tumor protein	TCTP/TPT1	-0.66	-1.58	0.00186	0.0221
29	Clusterin	Clusterin/CLU	-0.61	-1.53	0.00014	0.00344
30	Tissue Factor	TF/F3	-0.59	-1.51	4.00E-05	0.00141
31	Kunitz-type protease inhibitor 2	SPINT2/SPINT2	-0.6	-1.51	0.000404	0.00737
32	Glutathione S-transferase A3	GSTA3/GSTA3	-0.6	-1.51	0.00548	0.0487
33	NudC domain-containing protein 3	NUDC3/NUDCD3	-0.59	-1.5	1.57E-06	0.000118
34	Cytoplasmic protein NCK1	NCK1/NCK1	-0.59	-1.5	2.15E-06	0.000135

### Complement pathway and components were dysregulated in the HS transcriptomes and blood proteome

**Fig 2:**
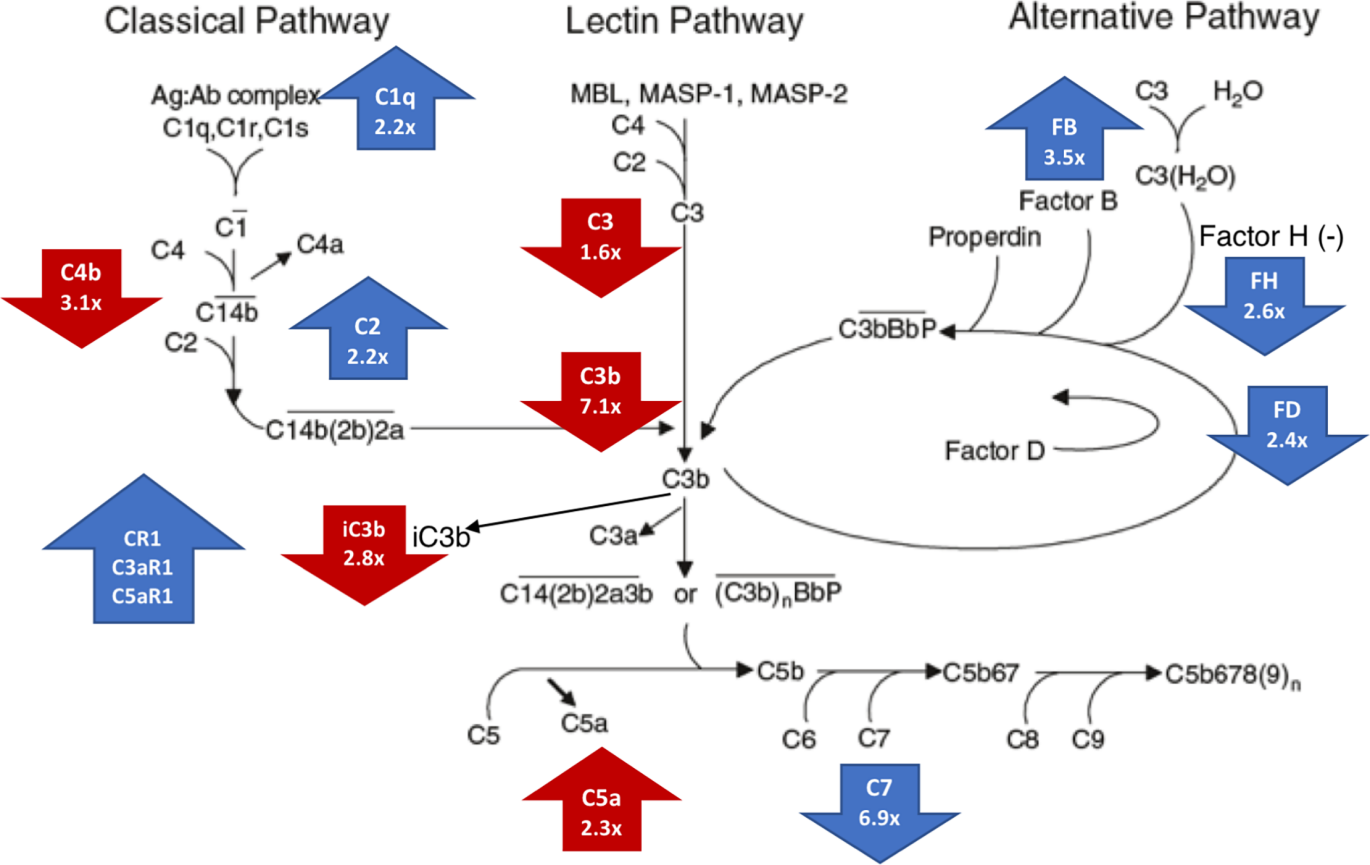
Dysregulation of complement pathway components. Fold change of skin DEGs (S4 Table) (blue arrows) and blood DEPs (Table 1) (red arrows) in the context of the complement cascade. It is not entirely clear which of the three complement activation pathways is engaged in HS, classical, lectin or alternative pathways. However, the classical pathway may be primarily implicated because of the involvement of C4 and the presence of immunoglobulins in the skin and blood. Additionally, lectin pathway activators, such as MBL and MASP, were not identified as DEGs in the skin or DEPs in the serum. Reprinted from Springe Nature under a CC BY license, with permission from Springe Nature, original copyright 2008. Modified by permission from Springer Customer Service Center GmbH: Springer Nature, Clinical and Basic Immunodermatology by Gaspari and Tyring, COPYRIGHT (2008).

*Complement* was a commonly identified Hallmark pathway in the HS skin and blood transcriptomes and HS blood proteome (S1 File Pages B, D and F). In the context of the classical complement cascade, C5a was upregulated and C4b, C3, C3b and iC3b were downregulated in the HS blood proteome (Fig 2). In the skin transcriptome, C1q and C2 were elevated and C7 was decreased. Upregulated skin DEGs also included complement receptors CR1, C3aR1 and C5aR1. In the alternative complement pathway, Factor B was elevated, Factor D was reduced, and inhibitor Factor H was reduced in the skin. C3 and C5 were correlated with disease severity (p<0.1) (S1 File Page J). The relationships between candidate serum biomarkers identified in the literature, and the skin transcriptome and blood proteome are discussed further in S1 Text and presented in S8 Table.

## Discussion

**Fig 3:**
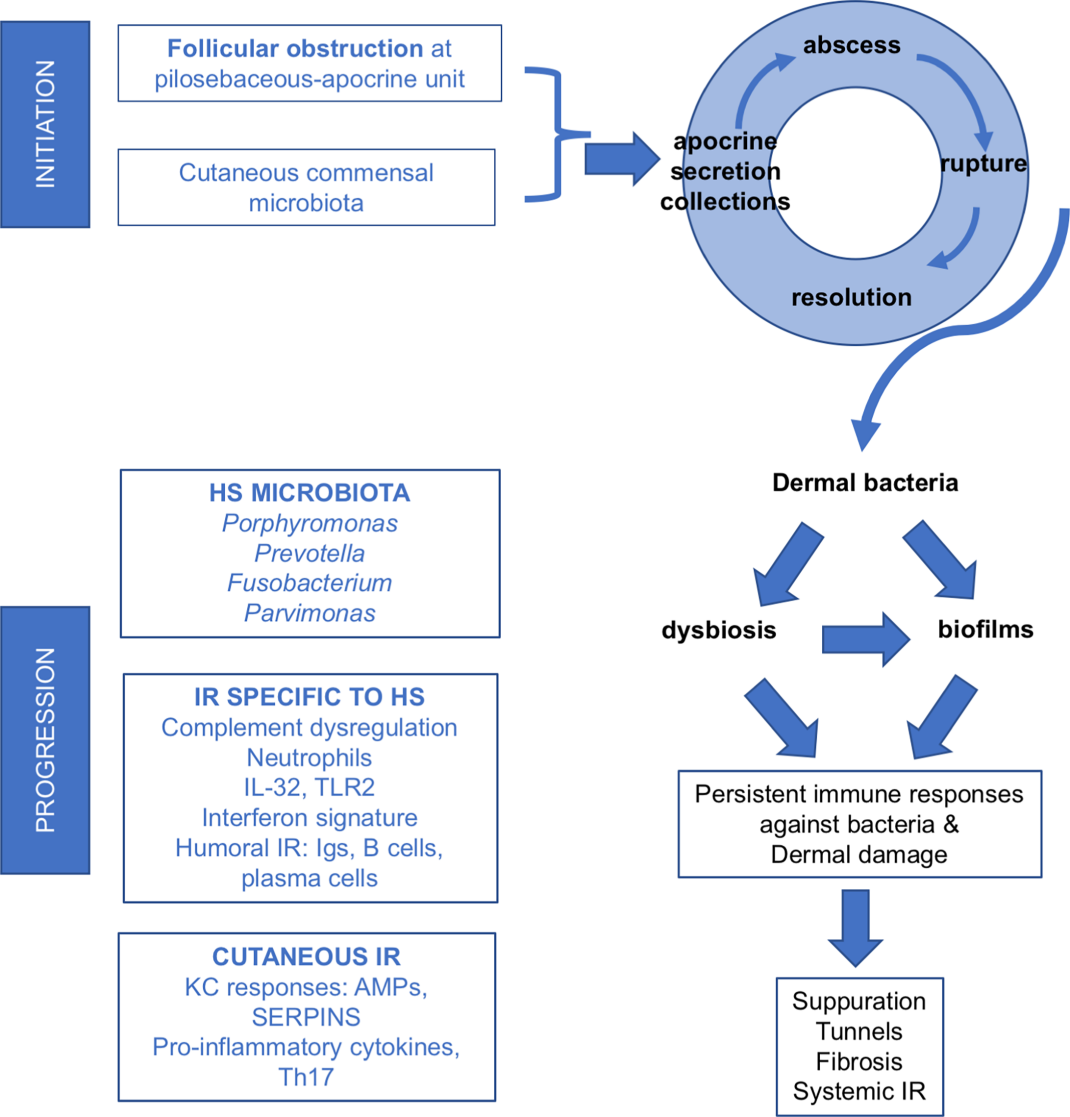
Proposed pathogenesis of hidradenitis suppurativa. Pathogenesis of hidradenitis suppurativa (HS) can be considered in two phases, initiation and progression. Our analysis of advanced HS showed immunoglobulin (Igs) transcripts, antimicrobial peptides (AMPs), an interferon signature, plasma cells in lesional skin, and an association with complement dysregulation. Considered in the context of prior findings in HS such as numerous neutrophils, B cells, plasma cells, TLR2, and IL-32, there appears to be a concerted immune response (IR) to eradicate bacteria in HS. These efforts may drive HS disease progression. There are also data supporting general cutaneous immune responses such as abundant AMPs, pro-inflammatory cytokines including IL-1, IL-6, IL-17, IL-12/23, and a dysregulated Th17/T-reg cell axis (often seen in other chronic skin diseases such as psoriasis or atopic dermatitis).

Much remains to be learned about the cause of HS, and *omics* platforms provide extensive survey-type tools to unravel disease pathways and generate hypotheses. Our main findings in HS lesional skin were abundant immunoglobulin genes, AMP's, an interferon signature, and plasma cells. In this cohort of moderate-to-severe HS patients, the complement pathway was dysregulated in both the skin and blood. In the blood, there was an increase in C5a, with a reduction of components in the proximal part of the complement pathway (C3, C4, and iC3b). These new data are incorporated into a proposed pathogenic model in Fig 3 with pathogenic elements unique to HS and those shared with other chronic skin diseases.

While many inflammatory cell types have been identified in HS lesions, the most abundant were neutrophils and aggregates of CD20^+^ B cells [18], and CD138^+^ plasma cells have also been observed [19]. A polyclonal hyperglobulinemia in HS has been reported [20] which may be explained by the presence of cutaneous plasma cells. Our findings confirm that plasma cells and levels of immunoglobulin genes may be very abundant in HS, possibly to opsonize bacteria. The differential activation of interferon gene-sets also supports an anti-microbial host response in the skin. However, the role of the humoral immune system in HS has not yet been well characterized. There is a case report of an anti-B cell therapy being effective in HS [21].

Recently there has been a new appreciation for the functions of complement, one of the most evolutionarily conserved elements of the innate immune system [22, 23]. Complement has also been shown to be very important in maintenance of normal cutaneous commensal bacterial function [24]. Recently, serum complement has also shown to be elevated in HS [25] and may present a novel therapeutic target [26].

Complement dysregulation and plasma cells observed in HS may indicate a critical role of bacteria in HS disease progression. Bacterial culture, and more recently metagenomic analysis, have identified that most of the bacteria present in HS are normal commensal microbiota. *Porphyromonas* and *Prevotella* taxa, gram-negative anaerobic rods, appear to be abundant in HS lesions [27, 28]. *Porphyromonas gingivalis* has been studied in the context of periodontitis, and is considered a “keystone pathogen” in that disease [29]. The dominance of *Porphyromonas gingivalis* leads to a reduction of other commensal bacteria or “dysbiosis”. One of the main mechanisms it utilizes to activate the immune system is via complement. Other elements of the host immune system are then recruited to try to eradicate these bacteria, including neutrophils, plasma cells and immunoglobulins.

Both *omics* and candidate approaches have their limitations: both are dependent upon the unique probes that are selected, and some cytokines may need more sensitive detection methods than the *omics* platforms [13]. Individual cytokines have been shown to be elevated in the serum of HS patients in some studies, including TNF [30], IL-17A [31], IL-6 [32, 33], and IL-32 [34]. Dysregulation of the Th17/Treg axis has been demonstrated in HS [35], and the “keratinocyte + IL-17” gene set was represented in the HS skin transcriptome in our analysis. IL-6 may be a driver of downstream systemic inflammation in HS inducing CRP, hepcidin and anaemia of chronic disease, immunoglobulins, Th17 differentiation, as well as contributing to other features seen in HS such as depression [36]. IL-32 is a novel cytokine produced by many immune cells, and was increased in HS compared to healthy controls, AD and PS [34]. Intracellular IL-32 has an important role in controlling intracellular infectious organisms, and secreted IL-32 can amplify immune responses by the induction of many cytokines including TNF, IL-1, IL-6, IL-23, IL-10, and Th1/17 cells [37, 38].

In summary, we have interpreted our findings of abundant immunoglobulin transcripts, AMPs, interferon signature, plasma cells, and complement dysregulation to indicate an effort by the immune system to control commensal bacteria in HS. Future investigations will test this hypothesis and hopefully lead to novel therapeutic approaches for this neglected disease.

## Conclusion

There is much to be learned by mining data from powerful “hypothesis-free” *omics* platforms. From a single cohort of HS patients in two studies [4, 6], we performed deep data analysis using advanced analytics such as GSVA and CIBERSORT. We identified abundant immunoglobulin transcripts, AMPs, an interferon signature, and plasma cells in HS lesions. There was also complement dysregulation in HS blood and skin. These findings suggest that HS disease is due to an exuberant inflammatory response which may be driven by dysbiotic commensal bacteria, and/or biofilms. Our findings led us to reconsider the role of bacteria in HS pathogenesis, and this may have important therapeutic implications.

## Supporting information

S1 Text Additional results from analysis. (PDF)

S1 File **Pages A-K**.
**Page A. Overview of analysis and integration of data, pathway analysis, and cellular composition predictive modeling.** The data for analysis was from 17 patients (pt) with moderate-to-severe hidradenitis suppurativa (HS), with matching clinical data, lesional skin (n = 17) and non-lesional skin (n = 13), and blood pre-treatment (pre) and post-treatment (post) with ustekinumab (described further in S2 Table). There were two different groups of ten healthy volunteers for blood samples (a and b). Fold change (FCH) for each analysis is provided as supplemental data for future analyses. * Prior analysis published as list of differentially expressed genes (DEGs) (in Blok JL et al. 2016, ref [4]). Skin DEGs with FCH>2.0 and FDR<0.05 (“all”) were filtered for a list of unique transcripts (”unique”). Differentially expressed proteins (DEPs; FCH>1.5, FDR<0.05) define the *HS disease proteome* (“all”, 62 DEPs), and comparison with the proteome of other chronic skin diseases defines the *unique HS proteomic signature* (“unique” 16 DEPs). Gene Set Variation Analysis (GSVA) of Hallmark and other curated gene-sets, including psoriasis and atopic dermatitis was conducted. CIBERSORT is a platform to enumerate cellular composition from gene expression (Newman et al. 2015).
**Page B. Hallmark gene-sets in HS skin transcriptome.** Heatmap showing Gene Set Variation Analysis (GSVA) scores of Hallmark gene-sets with differential activation in lesional (LS) versus non-lesinal (NL) skin (FDR <0.05) (S4 Table).
**Page C. Changes in HS, psoriasis (PS) and atopic dermatitis (AD) and associated gene-sets in the HS skin transcriptome.** Bar plots showing z-score log-fold change (+/-SD) of HS, PS and AD gene-sets in lesional (LS) versus non-lesional (NL) HS skin. Z-score changes for (A) gene-sets that are up-regulated and (B) down-regulated in HS, PS and AD. Asterix indicates significant difference in z-score LSvsNL. Gene-sets: Nicastrin knockout (NCSTN), macrophages + interferon (MACS+IFN), tissue IFN signature (IFN227), keratinocytes + IL-17 (KC+IL-17), keratinocytes + TNF (KC+TNF), psoriasis (PS), atopic dermatitis (AD). References for each gene-set are in the text. (***FDR<0.001).
**Page D. Hallmark gene-sets in HS blood transcriptome.** Heatmap showing Gene Set Variation Analysis (GSVA) scores of Hallmark gene-sets with differential activation in HS blood transcriptome versus normal volunteers (FDR <0.05) (S5 Table).
**Page E. CIBERSORT analysis of HS blood transcriptome.** (A) Heatmap showing normalized absolute abundance for each cell type in individual samples, mean abundance for each cell type is also displayed (left, green); (B) bar graph showing absolute abundance of each cell type in Normal and HS samples; and (C) mean abundance of each cell type in all Normal samples (left) and HS samples (right). Data from S5 Table. There were no significant differences in proportion of cell types in this analysis.
**Page F. Expression of HS blood proteome in HS patients, at baseline and after treatment with ustekinumab, and healthy volunteers.** Heatmap showing differentially expressed proteins (DEPs) in HS versus normal volunteers (FCH>1.5, FDR<0.05), also listed in Table 2. None of these DEPs changed significantly with ustekinumab treatment (FCH>1.5, FDR<0.05).
**Page G. Hallmark gene-sets in HS blood proteome.** Heatmap showing Gene Set Variation Analysis (GSVA) scores of Hallmark gene/protein-sets with differential activation in the blood proteome of HS patients versus normal volunteers (FDR <0.05) (S6 Table).
**Page H. Determining the unique HS proteomic signature.** (A) Approach to determine unique HS proteomic signature compared to other skin diseases (from Wang *et al*, 2017). (B) Unique upregulated (green) and downregulated (red) differentially expressed proteins (DEPs) in moderate- to-severe hidradenitis suppurativa (HS) compared to atopic dermatitis (AD), psoriasis (PS), and contact dermatitis (CD) (FCH>1.5, FDR<0.05). (C) Unique genes of AD, PS, and CD (green) and shared upregulated genes of the three diseases (white). Of the six DEPs shared between AD, PS and CD, only C5a was upregulated across all four diseases. For AD, PS, and CD, DEPs that were identified as unique for each of these three diseases were not upregulated DEPs in HS vs normal.
**Page I. Analysis of the HS differentially expressed proteins** (**DEPs) in patients treated with ustekinumab who did not respond.** Heatmap showing 4 differentially expressed proteins (DEPs) in HS (responders n = 14; non-responders, Non-Res, n = 3) versus normal volunteers (n = 10) (FCH>1.5, FDR <0.05). In the comparison of pre-treatment (Pre) and post-treatment (Post) in non-responders, there were 4 HS DEPs that were differentially expressed on patients that did not respond to the treatment (FCH>1.5, FDR<0.05).
**Page J. Complement versus HS disease severity.** Scatter plots of disease activity in a cohort of HS patients (Hurley stage II and III) measured by modified Sartorius Score (mSS, range for these patients 8–241) versus complement differentially expressed proteins (DEPs). The patients were part of an ustekinumab clinical trial in HS, and serum proteins were measured at baseline (week 0) and after treatment (week 40). Normal volunteers are indicated with mSS = 0, but not included in the regression analysis. (A) serum C3, (B) serum C4, (C) serum C5 protein levels. Blue lines represent the linear fit for mSS score and expression/z-score, Pearson correlation coefficients (*r*) and p-values (*p*) are shown.
**Page K. Differentially expressed genes in HS lesional skin versus normal skin and status in HS lesional versus non-lesional skin.** Raw gene expression data from skin of de-roofed lesions (n = 7) versus normal skin (n = 6) on Illumina Human HT12 V4 bead Array (Hotz et al, JID, 2016, ref 5) was reanalyzed using the same approaches described in materials and methods. P-values were corrected for multiple hypothesis testing and are presented with FDR values (significance level <0.05). The upregulated DEGs are listed, along with their status in lesional (LS) versus non-lesional (NL) skin (from S4 Table). Several genes did not have a corresponding target on the Affymetrix Chip.
(PDF)

S1 Table Summary of studies. (PDF)

S2 Table Summary of patients. (XLSX)

S3 Table Published IPA pathways. (PDF)

S4 Table HS skin transcriptome ALL.csv. (CSV)

S5 Table HS blood transcriptome ALL.csv. (CSV)

S6 Table HS blood proteome ALL.csv. (CSV)

S7 Table Blood proteome HS vs other skin diseases.csv. (CSV)

S8 Table Candidate biomarkers across platforms. (PDF)

## Abbreviations:

HS: hidradenitis suppurativa
DEGs: differentially expressed genes
DEPs: differentially expressed proteins
FCH: fold change
FDR: false discovery rate
LS: lesional
NL: non-lesional
GSVA: Gene Set Variation Analysis
AMPs: antimicrobial peptides
mSS: modified Sartorius score
PS: psoriasis
AD: atopic dermatitis
CD contact dermatitis
