# Supplementary material for: Integrating the skin and blood transcriptomes and serum proteome in hidradenitis suppurativa reveals complement dysregulation and a plasma cell signature
Source: PLoS One. 2018 Sep 28;13(9):e0203672. doi: 10.1371/journal.pone.0203672 (PMC6162087; doi:10.1371/journal.pone.0203672)
Supplement: S1 Text — (PDF) [file pone.0203672.s001.pdf]

Supporting Information
S1 Text

Title: Integrating the skin and blood transcriptomes and serum proteome in hidradenitis suppurativa reveals a complement and plasma cell signature.

Lauren K. Hoffman[1¶], Lewis E. Tomalin[2¶], Gregory Schultz[3], Michael D. Howell[4], Niroshana Anandasabapathy[5], Afsaneh Alavi[6], Mayte Suarez-Farinas[2], and Michelle A. Lowes[7*]

[1] Albert Einstein College of Medicine, Bronx, New York, New York, USA
[2] Department of Population Health and Science Policy, Icahn School of Medicine at Mount Sinai, New York, New York, USA
[3] Department of Obstetrics and Gynecology, University of Florida, Miami, Florida, USA
[4] Incyte Corporation, Wilmington, Delaware, USA
[5] Department of Dermatology, Weill Cornell Medical College, New York, New York, USA
[6] Department of Medicine, Division of Dermatology, Women's College Hospital, University of Toronto, Toronto, ON, Canada
[7] The Rockefeller University, New York, New York, USA

Results

Overview of studies and integrative approach

An overview of our integrative analysis is shown in S1 File A. Demographic features of this cohort of HS patients included female: male ratio of 13:4 and age range 20-53 years, which is similar to known demographic data for HS (S2 Table). The patients were in Hurley stage II (n=13) or stage III (n=4) and they were compared to healthy volunteers (n=10). Hurley staging is commonly used to define disease severity with mild disease limited to repeated inflammatory cystic nodules (stage I), that may be connected by isolated tunnels or tracts (stage II) or form a network of bridging dermal tunnels (stage III) associated with odiferous, purulent drainage. The top canonical Ingenuity pathways published from the original studies are listed in S3 Table [1-3].

Raw gene expression data from skin of de-roofed lesions (n=7) versus normal skin (n=6) on Illumina Human HT12 V4 bead Array [2] was reanalyzed using the same approaches described in materials and methods. P-values were corrected for multiple hypothesis testing and are presented with FDR values (significance level <0.05). The DEGs are listed in S1 File K, along with their status in lesional (LS) versus non-lesional (NL) skin (from S4 Table). Several genes did not have a corresponding target on the Affymetrix Chip.

Minimal differences were observed in the HS blood transcriptome

There were no changes in score activity for Interferon or nicastrin gene-sets in the HS blood transcriptome. The analysis of the cell composition in blood by CIBERSORT showed no significant difference in the abundance of immune cell types in the blood of HS patients (S1 File E).

HS blood proteome

To determine the unique HS proteomic signature, we compared HS Blood proteome with that of PS, AD, and CD, as outlined in S1 File H_A) [4]. Among the 62 HS DEPs, (Table 2), 36 were HS-specific. A new analysis was performed directly comparing the HS protein expression with that of PS, AD and CD (S7 Table). Of the 36/62 HS-specific DEPs, only 16 had significantly different expression in HS compared to all 3 diseases and thus defined the unique HS proteomic signature (S1 File H_B). Based on Wang et al., there were six proteins shared between AD, PS and CD compared to normal volunteers, but of these, only C5a was differentially expressed in HS (S1 File H_C). The unique upregulated DEPs for AD, PS, and CD, individually, were not upregulated in HS (S1 File H_C).

Response of HS blood proteome to ustekinumab treatment

In a 40-week study of ustekinumab (anti-IL-12/23) for patients with moderate-severe HS, responders were defined as individuals with a >30% improvement in modified Sartorius score (mSS; range for these patients 8-241) (S2 Table) [3]. MSS counts anatomical regions, type and number of lesions, extent of involvement and Hurley stage, into a single score [5]. Of the 62 DEPs in the HS blood proteome, none were significantly altered by treatment in HS patients (n=17) (post treatment versus pre-treatment, FCH>1.5) (S1 File F). No significant changes in DEPs were induced by treatment in responders (n=14). In the small group of non-responders (n=3), there were 4 DEGs that were differentially activated in pre-vs post treatment samples, namely C3, ICOS, Aflatoxin B1 Aldehyde Reductase Member 4 and PCSK7 (S1 File I). In GSVA analysis, no changes in any Hallmark gene-sets were induced by treatment in the whole group, responders or non-responders.

Relationship between candidate HS serum biomarkers and tissue gene expression

Candidate serum biomarkers were identified from the literature (S8 Table). Published serum biomarkers ESR [6], CRP [6, 7], sIL-2R [6, 8], TNF [9], sTNF-RII [10], IL-6 [10, 11], IL-17A [12], IL-32 [13], S100A8/A9 [8], lipocalin [14], chitinase-3-like protein (YKL-40) [15], MMP8 [16], IgA, IgE, and IgG [17], were increased in HS compared to normal individuals. In these publications, ESR, CRP, sIL-2R, IL-17, IL-6, YKL-40, MMP8, IgA and IgG were also elevated in advanced HS. Tissue expression of these biomarkers in HS lesions is noted in S8 Table.

The expression of these 12 biomarkers in the new analysis of the HS skin transcriptome (S4 Table) and HS blood proteome (S6 Table) is listed in S8 Table. The candidate biomarkers published in these papers were not identified as DEPs using the SOMAscan platform, either because they were not represented on the platform, or they did not meet significance even before testing for multiplicity. The following candidate biomarkers were also found to be DEGs in the HS skin transcriptome: IL2Rg, IL-6, IL-32, S100A8 and A9, lipocalin, and YKL40. Serum IgA, IgE, IgG were found to be elevated in HS blood [17], and there were many DEGs for immunoglobulin transcripts in the HS skin transcriptome.

References

1. Blok JL, Li K, Brodmerkel C, Jonkman MF, Horvath B. Gene expression profiling of skin and blood in hidradenitis suppurativa. Br J Dermatol. 2016;174(6):1392-4. doi: 10.1111/bjd.14371. PubMed PMID: 26707687.

2. Hotz C, Boniotto M, Guguin A, Surenaud M, Jean-Louis F, Tisserand P, et al. Intrinsic defect in keratinocyte function leads to inflammation in Hidradenitis suppurativa. J Invest Dermatol. 2016. doi: 10.1016/j.jid.2016.04.036. PubMed PMID: 27206704.

3. Blok JL, Li K, Brodmerkel C, Horvatovich P, Jonkman MF, Horvath B. Ustekinumab in hidradenitis suppurativa: clinical results and a search for potential biomarkers in serum. Br J Dermatol. 2016;174(4):839-46. Epub 2015/12/08. doi: 10.1111/bjd.14338. PubMed PMID: 26641739.

4. Wang J, Suarez-Farinas M, Estrada Y, Parker ML, Greenlees L, Stephens G, et al. Identification of unique proteomic signatures in allergic and non-allergic skin disease. Clin Exp Allergy. 2017;47(11):1456-67. doi: 10.1111/cea.12979. PubMed PMID: 28703865.

5. Revuz J. [Modifications to the Sartorius score and instructions for evaluating the severity of suppurative hidradenitis]. Ann Dermatol Venereol. 2007;134(2):173-4. PubMed PMID: 17375017.

6. Matusiak L, Bieniek A, Szepietowski JC. Soluble interleukin-2 receptor serum level is a useful marker of hidradenitis suppurativa clinical staging. Biomarkers. 2009;14(6):432-7. doi: 10.1080/13547500903075218. PubMed PMID: 19627253.

7. Hessam S, Sand M, Gambichler T, Bechara FG. Correlation of inflammatory serum markers with disease severity in patients with hidradenitis suppurativa (HS). J Am Acad Dermatol. 2015;73(6):998-1005. Epub 2015/09/28. doi: 10.1016/j.jaad.2015.08.052. PubMed PMID: 26410359.

8. Wieland CW, Vogl T, Ordelman A, Vloedgraven HG, Verwoolde LH, Rensen JM, et al. Myeloid marker S100A8/A9 and lymphocyte marker, soluble interleukin 2 receptor: biomarkers of hidradenitis suppurativa disease activity? Br J Dermatol. 2013;168(6):1252-8. doi: 10.1111/bjd.12234. PubMed PMID: 23320892.

9. Matusiak L, Bieniek A, Szepietowski JC. Increased serum tumour necrosis factor-alpha in hidradenitis suppurativa patients: is there a basis for treatment with anti-tumour necrosis

factor-alpha agents? Acta Derm Venereol. 2009;89(6):601-3. doi: 10.2340/00015555-0749. PubMed PMID: 19997690.

10. Jimenez-Gallo D, de la Varga-Martinez R, Ossorio-Garcia L, Albarran-Planelles C, Rodriguez C, Linares-Barrios M. The Clinical Significance of Increased Serum Proinflammatory Cytokines, C-Reactive Protein, and Erythrocyte Sedimentation Rate in Patients with Hidradenitis Suppurativa. Mediators Inflamm. 2017;2017:2450401. doi: 10.1155/2017/2450401. PubMed PMID: 28769536; PubMed Central PMCID: PMCPMC5523401.

11. Xu H, Xiao X, He Y, Zhang X, Li C, Mao Q, et al. Increased serum interleukin-6 levels in patients with hidradenitis suppurativa. Postepy Dermatol Alergol. 2017;34(1):82-4. doi: 10.5114/ada.2017.65626. PubMed PMID: 28261036; PubMed Central PMCID: PMCPMC5329110.

12. Matusiak L, Szczech J, Bieniek A, Nowicka-Suszko D, Szepietowski JC. Increased interleukin (IL)-17 serum levels in patients with hidradenitis suppurativa: Implications for treatment with anti-IL-17 agents. J Am Acad Dermatol. 2016. doi: 10.1016/j.jaad.2016.10.042. PubMed PMID: 28041632.

13. Thomi R, Yerly D, Yawalkar N, Simon D, Schlapbach C, Hunger RE. Interleukin-32 is highly expressed in lesions of hidradenitis suppurativa. Br J Dermatol. 2017. doi: 10.1111/bjd.15458. PubMed PMID: 28301691.

14. Wolk K, Wenzel J, Tsaousi A, Witte-Handel E, Babel N, Zelenak C, et al. Lipocalin-2 is expressed by activated granulocytes and keratinocytes in affected skin and reflects disease activity in acne inversa / hidradenitis suppurativa. Br J Dermatol. 2017. doi: 10.1111/bjd.15424. PubMed PMID: 28256718.

15. Matusiak L, Salomon J, Nowicka-Suszko D, Bieniek A, Szepietowski JC. Chitinase-3-like Protein 1 (YKL-40): Novel Biomarker of Hidradenitis Suppurativa Disease Activity? Acta Derm Venereol. 2015;95(6):736-7. doi: 10.2340/00015555-2061. PubMed PMID: 25655759.

16. Tsaousi A, Witte E, Witte K, Rowert-Huber HJ, Volk HD, Sterry W, et al. MMP8 Is Increased in Lesions and Blood of Acne Inversa Patients: A Potential Link to Skin Destruction and Metabolic Alterations. Mediators Inflamm. 2016;2016:4097574. doi: 10.1155/2016/4097574. PubMed PMID: 27843200; PubMed Central PMCID: PMCPMC5097814.

17. Hoffman LK, Ghias M, Cohen SR, Lowes MA. Polyclonal hyperglobulinemia and elevated acute phase reactants in hidradenitis suppurativa. Br J Dermatol. 2017. doi: 10.1111/bjd.15958. PubMed PMID: 28886222.
