# Supplementary material for: Integrating the skin and blood transcriptomes and serum proteome in hidradenitis suppurativa reveals complement dysregulation and a plasma cell signature
Source: PLoS One. 2018 Sep 28;13(9):e0203672. doi: 10.1371/journal.pone.0203672 (PMC6162087; doi:10.1371/journal.pone.0203672)
Supplement: S1 File — Pages A-K. Page A. Overview of analysis and integration of data, pathway analysis, and cellular composition predictive modeling. The data for analysis was from 17 patients (pt) with moderate-to-severe hidradenitis suppurativa (HS), with matching clinical data, lesional skin (n = 17) and non-lesional skin (n = 13), and blood pre-treatment (pre) and post-treatment (post) with ustekinumab (described further in S2 Table). There were two different groups of ten healthy volunteers for blood samples (a and b). Fold change (FCH) for each analysis is provided as supplemental data for future analyses. * Prior analysis published as list of differentially expressed genes (DEGs) (in Blok JL et al. 2016, ref [4]). Skin DEGs with FCH>2.0 and FDR<0.05 (“all”) were filtered for a list of unique transcripts (”unique”). Differentially expressed proteins (DEPs; FCH>1.5, FDR<0.05) define the HS disease proteome (“all”, 62 DEPs), and comparison with the proteome of other chronic skin diseases defines the unique HS proteomic signature (“unique” 16 DEPs). Gene Set Variation Analysis (GSVA) of Hallmark and other curated gene-sets, including psoriasis and atopic dermatitis was conducted. CIBERSORT is a platform to enumerate cellular composition from gene expression (Newman et al. 2015). Page B. Hallmark gene-sets in HS skin transcriptome. Heatmap showing Gene Set Variation Analysis (GSVA) scores of Hallmark gene-sets with differential activation in lesional (LS) versus non-lesinal (NL) skin (FDR <0.05) (S4 Table). Page C. Changes in HS, psoriasis (PS) and atopic dermatitis (AD) and associated gene-sets in the HS skin transcriptome. Bar plots showing z-score log-fold change (+/-SD) of HS, PS and AD gene-sets in lesional (LS) versus non-lesional (NL) HS skin. Z-score changes for (A) gene-sets that are up-regulated and (B) down-regulated in HS, PS and AD. Asterix indicates significant difference in z-score LSvsNL. Gene-sets: Nicastrin knockout (NCSTN), macrophages + interferon (MACS+IFN), tissu [file pone.0203672.s002.pdf]

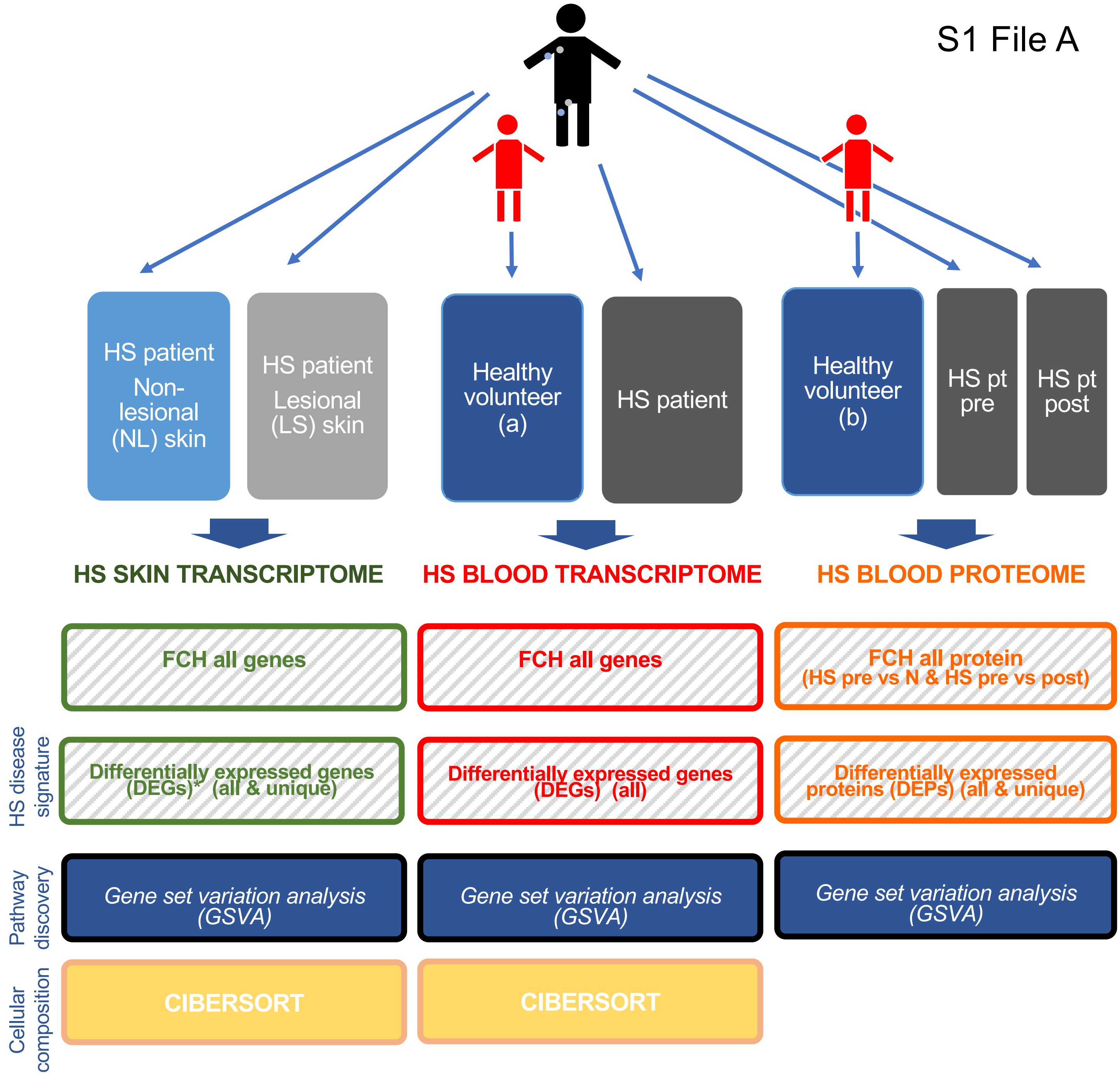
S1 File A
HS patient
Non-lesional (NL) skin
HS patient
Lesional (LS) skin
Healthy volunteer (a)
HS patient
Healthy volunteer (b)
HS pt pre
HS pt post
HS SKIN TRANSCRIPTOME
HS BLOOD TRANSCRIPTOME
HS BLOOD PROTEOME
FCH all genes
FCH all genes
FCH all protein (HS pre vs N & HS pre vs post)
HS disease signature
Differentially expressed genes (DEGs)* (all & unique)
Differentially expressed genes (DEGs) (all)
Differentially expressed proteins (DEPs) (all & unique)
Pathway discovery
Gene set variation analysis (GSVA)
Gene set variation analysis (GSVA)
Gene set variation analysis (GSVA)
Cellular composition
CIBERSORT
CIBERSORT

S1 File B

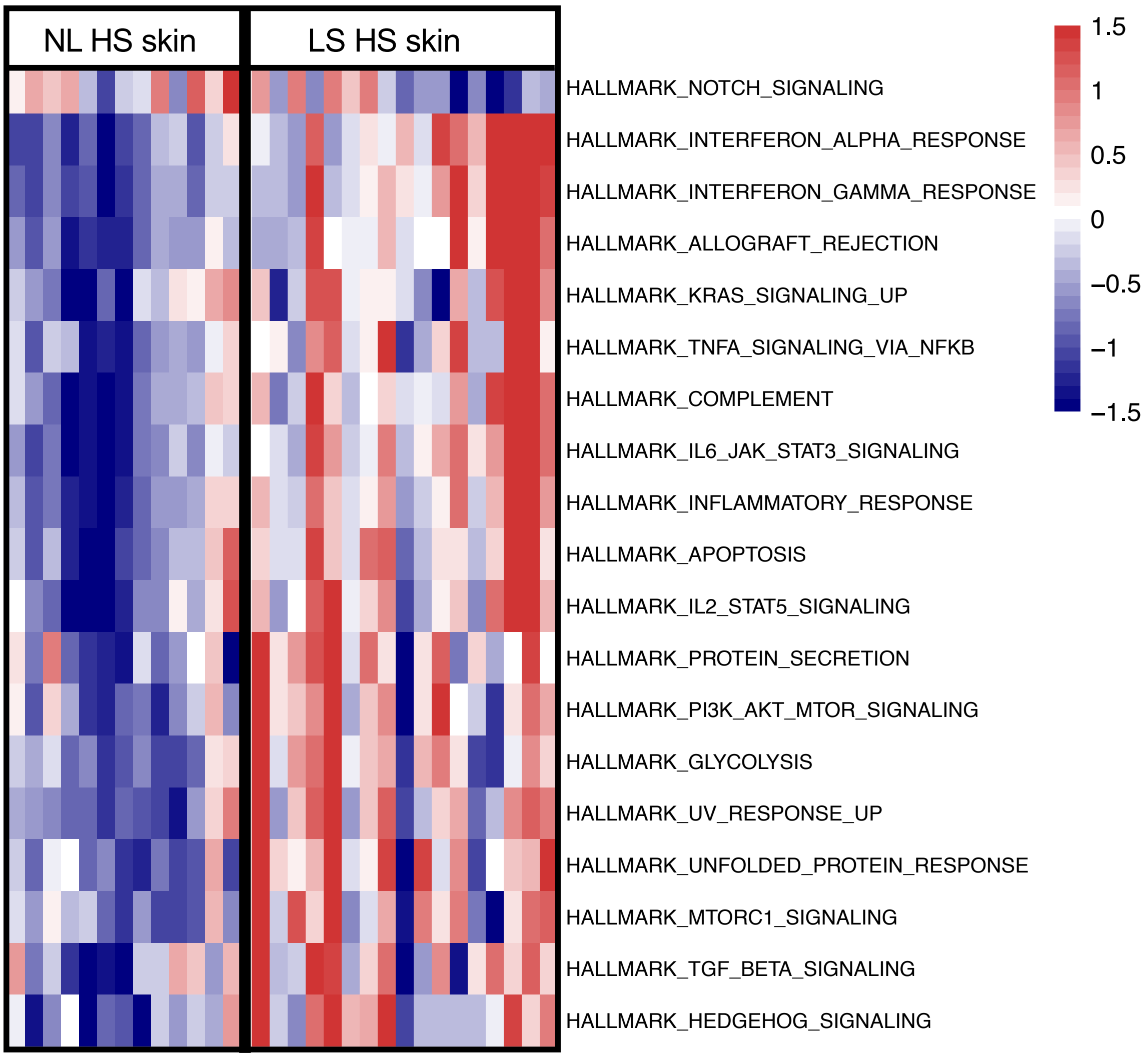
NL HS skin
LS HS skin
HALLMARK_NOTCH_SIGNALING
HALLMARK_INTERFERON_ALPHA_RESPONSE
HALLMARK_INTERFERON_GAMMA_RESPONSE
HALLMARK_ALLOGRAFT_REJECTION
HALLMARK_KRAS_SIGNALING_UP
HALLMARK_TNFA_SIGNALING_VIA_NFKB
HALLMARK_COMPLEMENT
HALLMARK_IL6_JAK_STAT3_SIGNALING
HALLMARK_INFLAMMATORY_RESPONSE
HALLMARK_APOPTOSIS
HALLMARK_IL2_STAT5_SIGNALING
HALLMARK_PROTEIN_SECRETION
HALLMARK_PI3K_AKT_MTOR_SIGNALING
HALLMARK_GLYCOLYSIS
HALLMARK_UV_RESPONSE_UP
HALLMARK_UNFOLDED_PROTEIN_RESPONSE
HALLMARK_MTORC1_SIGNALING
HALLMARK_TGF_BETA_SIGNALING
HALLMARK_HEDGEHOG_SIGNALING
1.5
1
0.5
0
−0.5
−1
−1.5

S1 File C

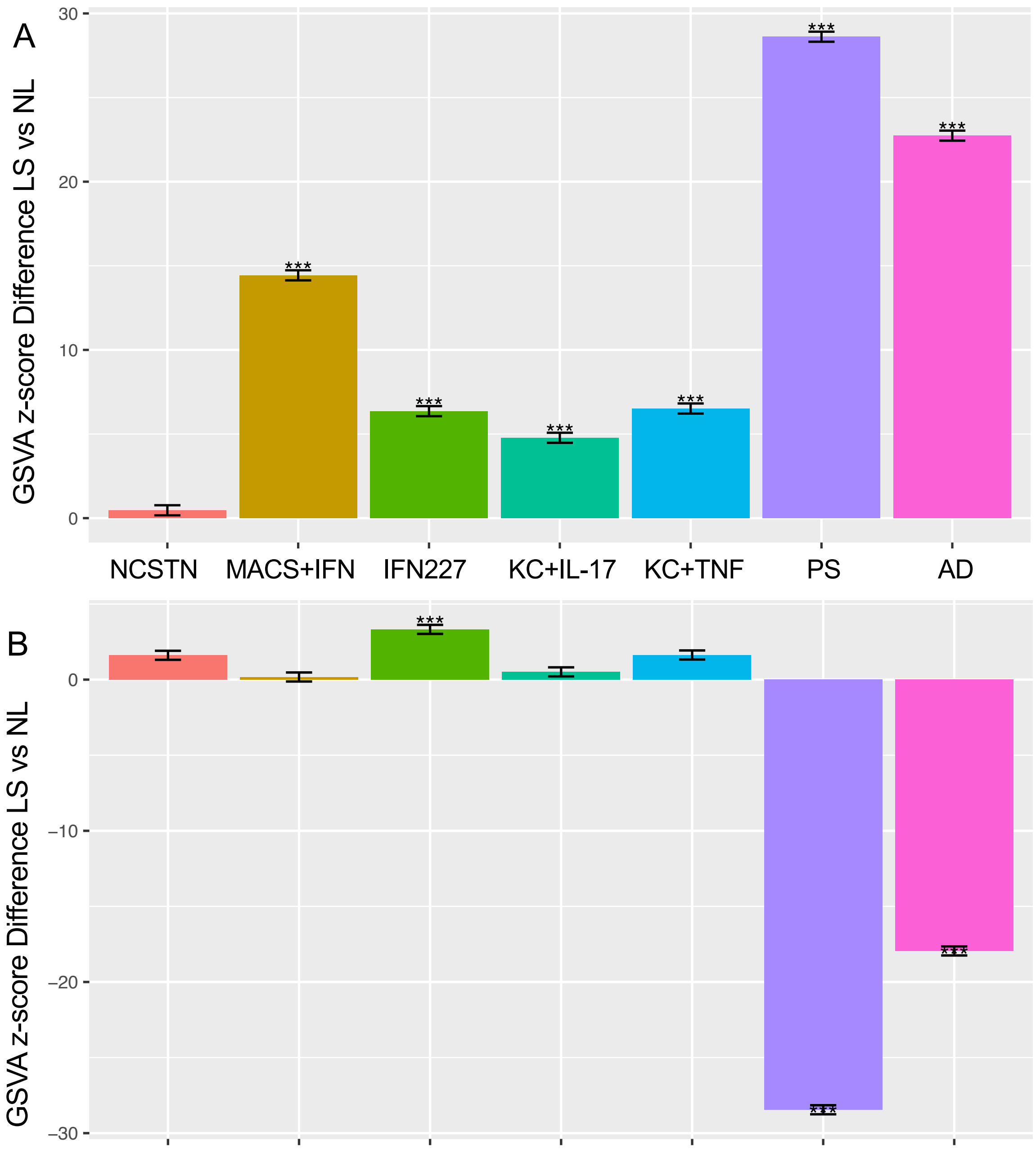
A
B
GSVA z-score Difference LS vs NL
30
20
10
0
-10
-20
-30
***
NCSTN
MACS+IFN
IFN227
KC+IL-17
KC+TNF
PS
AD

S1 File D

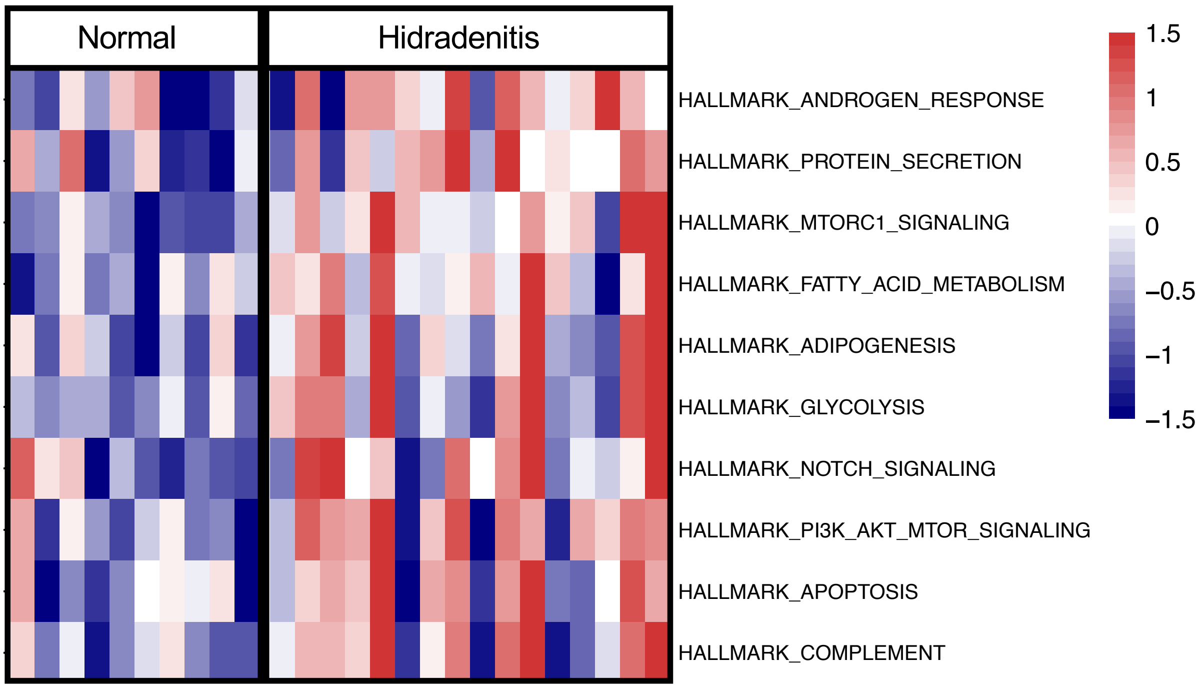

Normal
Hidradenitis
HALLMARK_ANDROGEN_RESPONSE
HALLMARK_PROTEIN_SECRETION
HALLMARK_MTORC1_SIGNALING
HALLMARK_FATTY_ACID_METABOLISM
HALLMARK_ADIPOGENESIS
HALLMARK_GLYCOLYSIS
HALLMARK_NOTCH_SIGNALING
HALLMARK_PI3K_AKT_MTOR_SIGNALING
HALLMARK_APOPTOSIS
HALLMARK_COMPLEMENT
1.5
1
0.5
0
−0.5
−1
−1.5

S1 File E

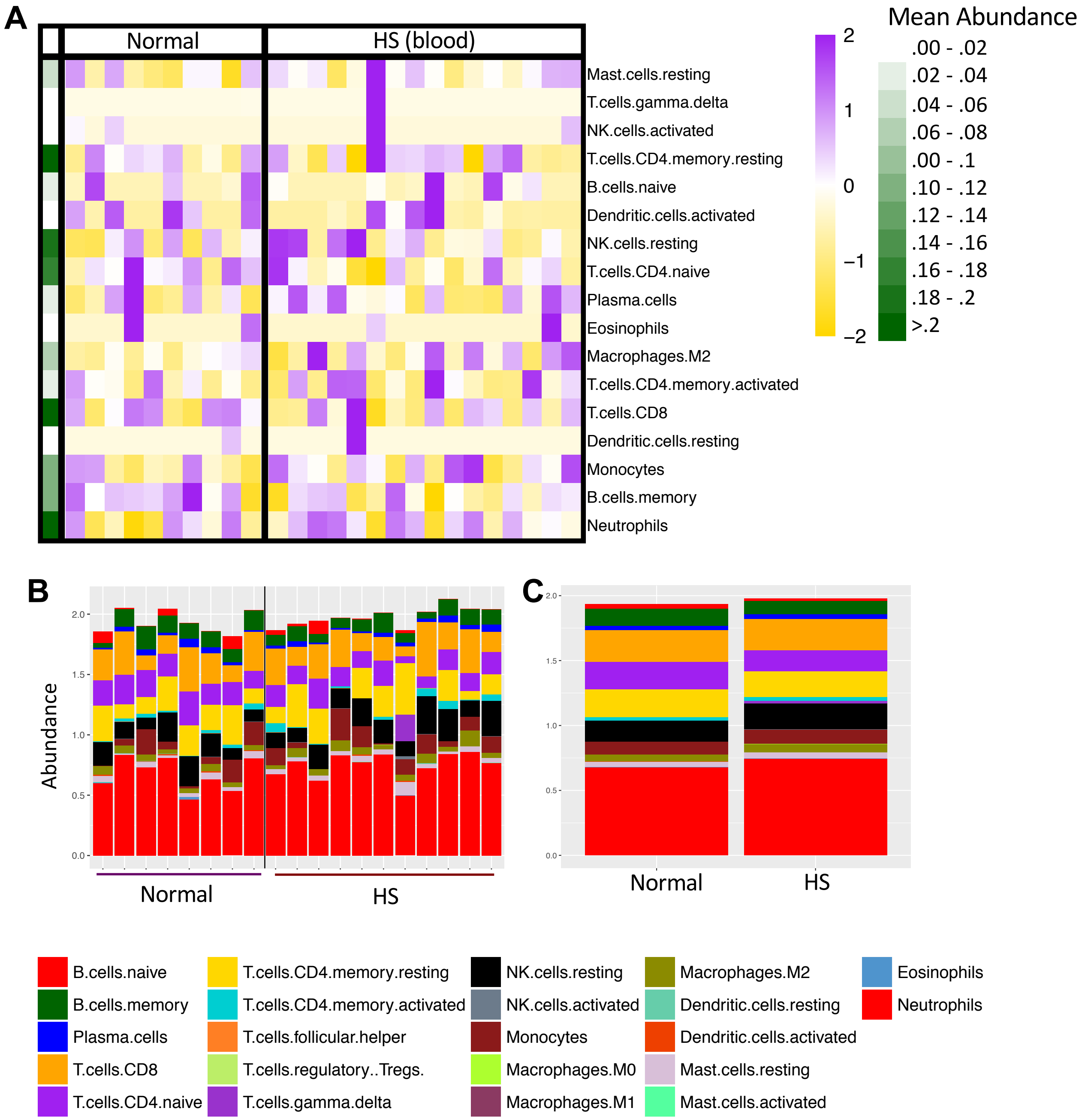

A
Normal
HS (blood)
Mast.cells.resting
T.cells.gamma.delta
NK.cells.activated
T.cells.CD4.memory.resting
B.cells.naive
Dendritic.cells.activated
NK.cells.resting
T.cells.CD4.naive
Plasma.cells
Eosinophils
Macrophages.M2
T.cells.CD4.memory.activated
T.cells.CD8
Dendritic.cells.resting
Monocytes
B.cells.memory
Neutrophils
2
1
0
−1
−2
Mean Abundance
.00 - .02
.02 - .04
.04 - .06
.06 - .08
.00 - .1
.10 - .12
.12 - .14
.14 - .16
.16 - .18
.18 - .2
>.2
B
Abundance
2.0
1.5
1.0
0.5
0.0
Normal
HS
C
2.0
1.5
1.0
0.5
0.0
Normal
HS
B.cells.naive
B.cells.memory
Plasma.cells
T.cells.CD8
T.cells.CD4.naive
T.cells.CD4.memory.resting
T.cells.CD4.memory.activated
T.cells.follicular.helper
T.cells.regulatory..Tregs.
T.cells.gamma.delta
NK.cells.resting
NK.cells.activated
Monocytes
Macrophages.M0
Macrophages.M1
Macrophages.M2
Dendritic.cells.resting
Dendritic.cells.activated
Mast.cells.resting
Mast.cells.activated
Eosinophils
Neutrophils

S1 File F

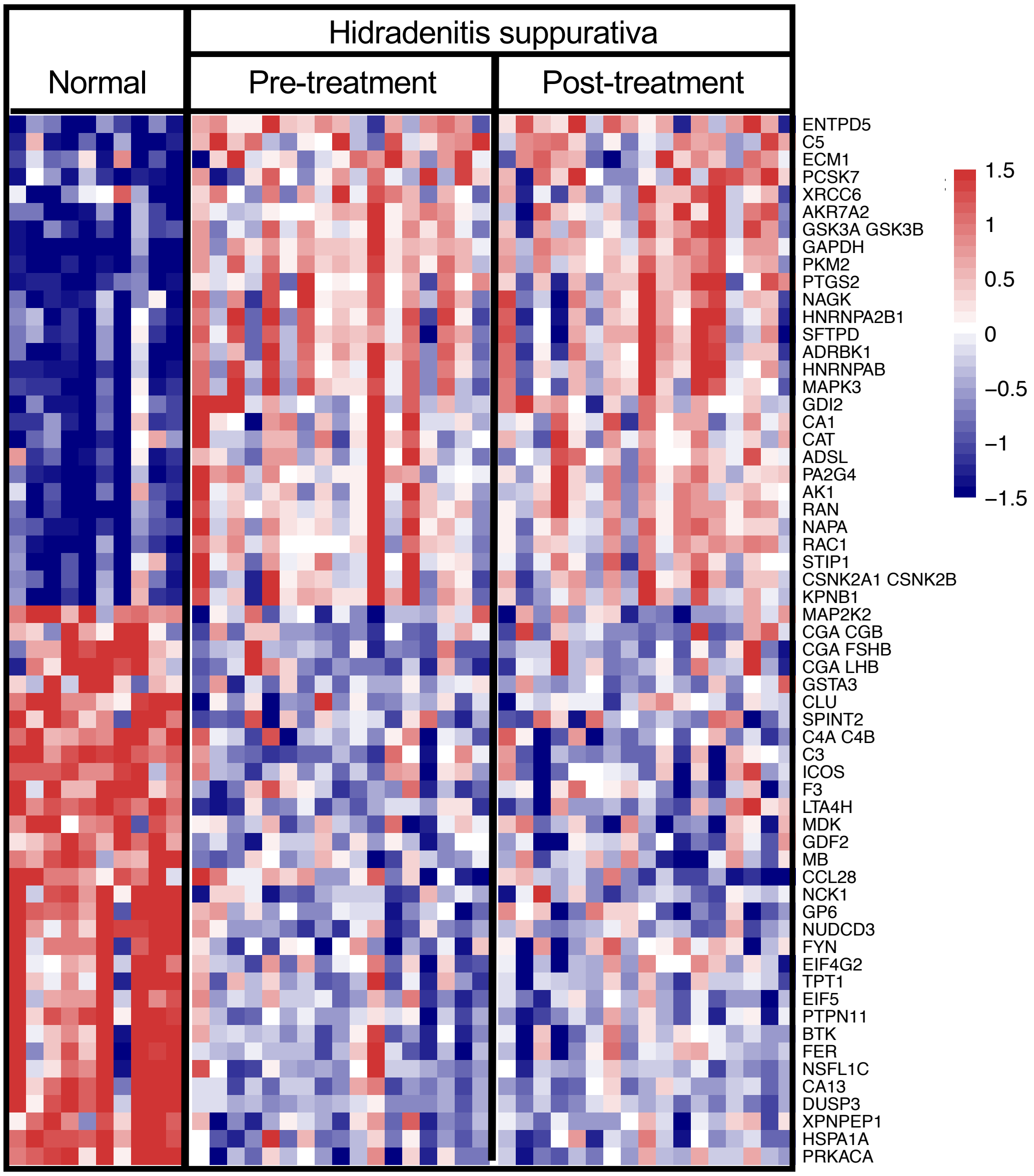

Hidradenitis suppurativa
Normal
Pre-treatment
Post-treatment
ENTPD5
C5
ECM1
PCSK7
XRCC6
AKR7A2
GSK3A GSK3B
GAPDH
PKM2
PTGS2
NAGK
HNRNPA2B1
SFTPD
ADRBK1
HNRNPAB
MAPK3
GDI2
CA1
CAT
ADSL
PA2G4
AK1
RAN
NAPA
RAC1
STIP1
CSNK2A1 CSNK2B
KPNB1
MAP2K2
CGA CGB
CGA FSHB
CGA LHB
GSTA3
CLU
SPINT2
C4A C4B
C3
ICOS
F3
LTA4H
MDK
GDF2
MB
CCL28
NCK1
GP6
NUDCD3
FYN
EIF4G2
TPT1
EIF5
PTPN11
BTK
FER
NSFL1C
CA13
DUSP3
XPNPEP1
HSPA1A
PRKACA
1.5
1
0.5
0
−0.5
−1
−1.5

S1 File G

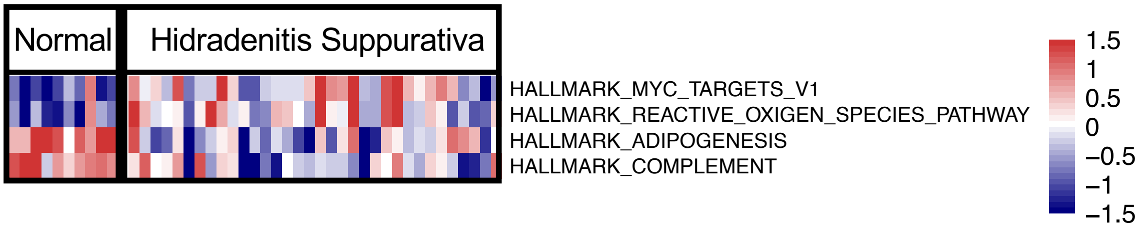
Normal
Hidradenitis Suppurativa
HALLMARK_MYC_TARGETS_V1
HALLMARK_REACTIVE_OXIGEN_SPECIES_PATHWAY
HALLMARK_ADIPOGENESIS
HALLMARK_COMPLEMENT
1.5
1
0.5
0
−0.5
−1
−1.5

S1 File H

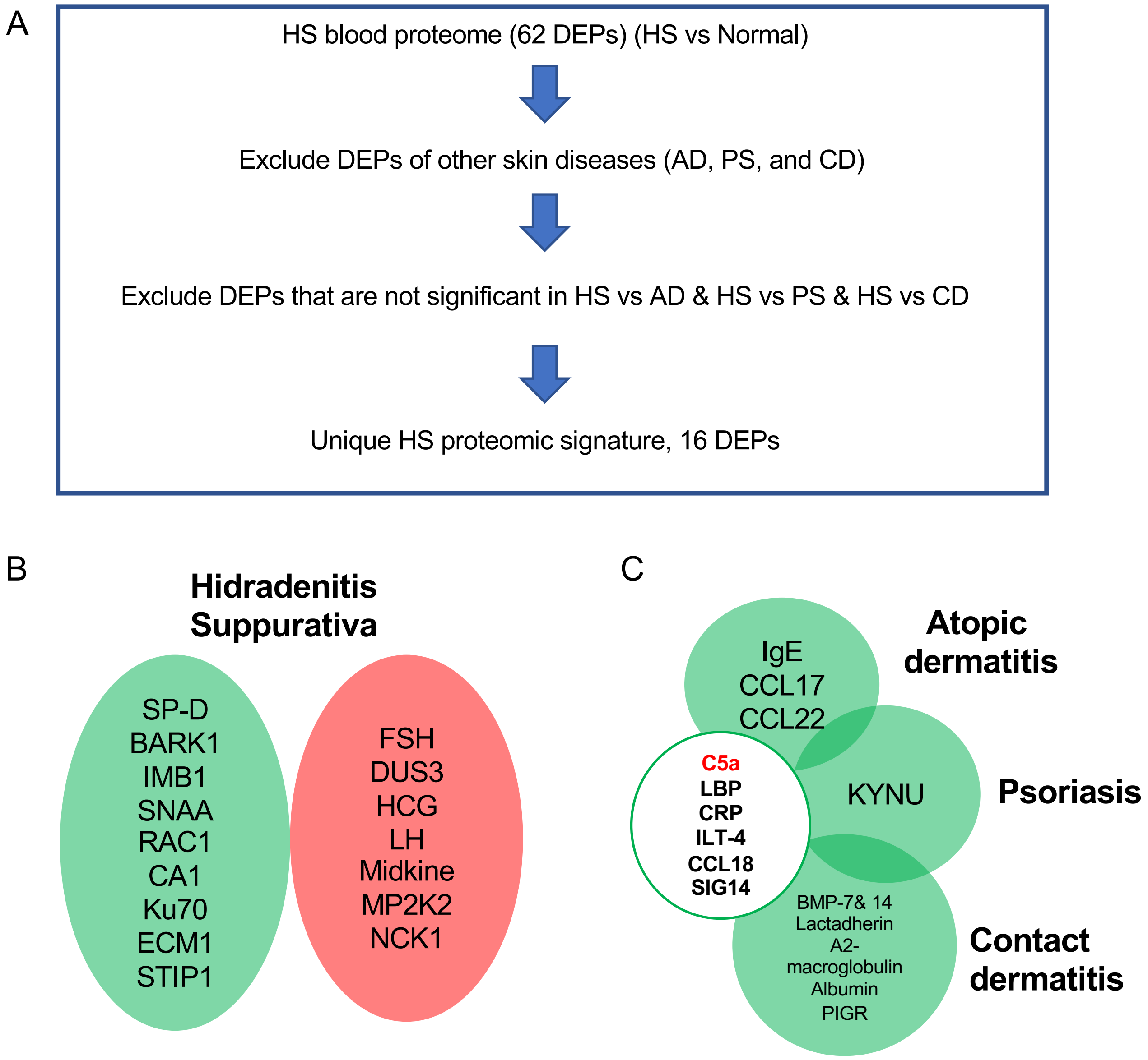
A
HS blood proteome (62 DEPs) (HS vs Normal)
Exclude DEPs of other skin diseases (AD, PS, and CD)
Exclude DEPs that are not significant in HS vs AD & HS vs PS & HS vs CD
Unique HS proteomic signature, 16 DEPs
B
Hidradenitis Suppurativa
SP-D
BARK1
IMB1
SNAA
RAC1
CA1
Ku70
ECM1
STIP1
FSH
DUS3
HCG
LH
Midkine
MP2K2
NCK1
C
Atopic dermatitis
IgE
CCL17
CCL22
Psoriasis
KYNU
Contact dermatitis
BMP-7& 14
Lactadherin
A2-macroglobulin
Albumin
PIGR
C5a
LBP
CRP
ILT-4
CCL18
SIG14

S1 File I

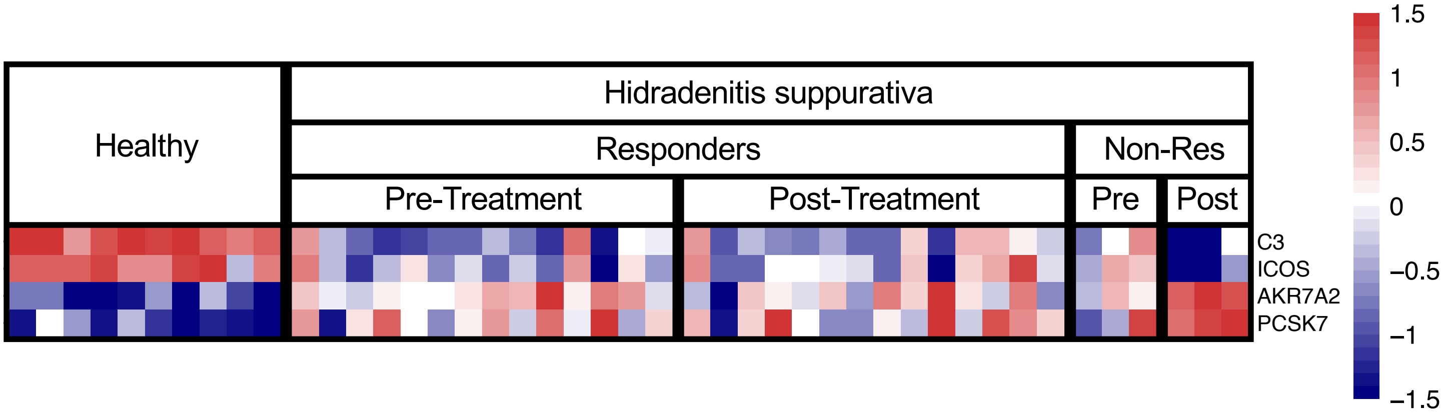
Hidradenitis suppurativa
Healthy
Responders
Non-Res
Pre-Treatment
Post-Treatment
Pre
Post
C3
ICOS
AKR7A2
PCSK7
1.5
1
0.5
0
−0.5
−1
−1.5

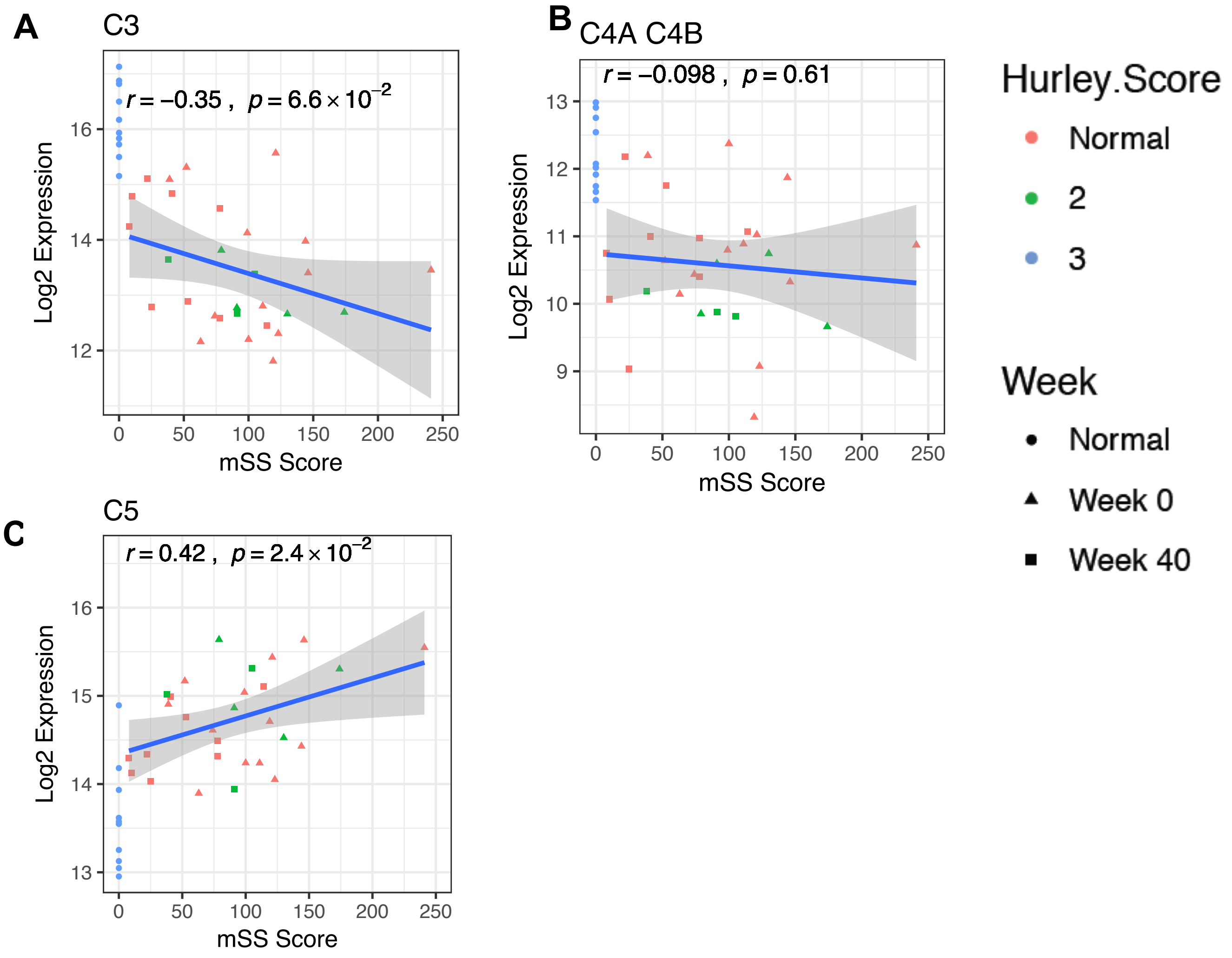
A
C3
r = −0.35 , p = 6.6 × 10−2
Log2 Expression
mSS Score
B
C4A C4B
r = −0.098 , p = 0.61
Log2 Expression
mSS Score
C
C5
r = 0.42 , p = 2.4 × 10−2
Log2 Expression
mSS Score
Hurley.Score
Normal
2
3
Week
Normal
Week 0
Week 40

Gene Symbol	logFCH LSvsN	FCH LSvsN	Pval LSvsN	FDR LsvsN	StatusFCH2 FDR0.05 LSvsN	status LSvsNL
S100A7	4.16	17.89	7.15E-06	0.0342	1	1
PI3	4.08	16.97	2.52E-06	0.0342	1	1
S100A9	3.61	12.18	1.60E-06	0.0342	1	1
SPRR2F	3.37	10.32	1.80E-05	0.0342	1	not on chip
AKR1B10	3.16	8.92	2.89E-05	0.0342	1	1
SPRR2B	2.97	7.85	3.21E-05	0.0349	1	not on chip
S100A8	2.89	7.39	4.62E-06	0.0342	1	1
SPRR2D	2.86	7.24	6.65E-06	0.0342	1	1
KRT16	2.66	6.32	2.39E-05	0.0342	1	1
SPRR2C	2.65	6.27	6.77E-05	0.0399	1	1
LOC729252	2.27	4.82	4.05E-05	0.0369	1	not on chip
LOC400578	2.2	4.58	3.93E-05	0.0369	1	not on chip
IFI27	2.18	4.53	1.46E-06	0.0342	1	1
GJB2	2.03	4.09	8.40E-06	0.0342	1	1
THY1	1.97	3.9	2.85E-05	0.0342	1	1
GJB6	1.67	3.18	6.87E-05	0.0399	1	0
CCL19	1.57	2.98	1.84E-05	0.0342	1	0
CFB	1.53	2.89	4.67E-05	0.0381	1	0
ZC3H12A	1.39	2.63	2.48E-05	0.0342	1	0
KRT6B	1.31	2.49	6.60E-05	0.0399	1	0
GGCT	1.13	2.18	7.12E-05	0.0399	1	0
CTSC	1.12	2.17	6.49E-05	0.0399	1	1
LY96	1.09	2.12	2.20E-05	0.0342	1	1
TNC	1.08	2.11	9.85E-06	0.0342	1	0
FKBP11	1.02	2.02	1.29E-05	0.0342	1	0
TYMP	1.01	2.02	2.40E-05	0.0342	1	0
BMP4	-1.01	-2.01	3.78E-05	0.0365	-1	0
BMP2	-1.03	-2.04	7.78E-05	0.0419	-1	0
WDR72	-1.1	-2.15	4.86E-06	0.0342	-1	-1
CLDN23	-1.11	-2.16	5.97E-05	0.0399	-1	-1
H3F3B	-1.12	-2.17	8.77E-06	0.0342	-1	-1
C9ORF61	-1.18	-2.26	4.42E-05	0.0379	-1	not on chip
FOXQ1	-1.2	-2.3	6.22E-05	0.0399	-1	-1
BTC	-1.55	-2.92	6.27E-05	0.0399	-1	-1
