# Supplementary material for: Integrating the skin and blood transcriptomes and serum proteome in hidradenitis suppurativa reveals complement dysregulation and a plasma cell signature
Source: PLoS One. 2018 Sep 28;13(9):e0203672. doi: 10.1371/journal.pone.0203672 (PMC6162087; doi:10.1371/journal.pone.0203672)
Supplement: S1 Table — (PDF) [file pone.0203672.s003.pdf]

S1 Table. Summary of Studies

	Study	Blood Samples	Skin biopsy	Analysis Platforms	Significance
1	Open label clinical trial of ustekinumab treatment for moderate-to-severe HS [1]	[%] Serum HS=17 moderate-severe HS; HC n=10		SOMAscan platform, 1129 serum proteins	DEPs FCH>1.5 FDR<0.05
2	Research Letter of gene expression profiling of skin and blood in HS [2]	Whole Blood (WB) HS=17 moderate-severe HS; HC n=10	[#] LS 3mm skin biopsy n=17, NL n=13 from adjacent normal appearing skin	Microarray on HG-U133+ PM Array (Affymetrix)<br><br>*GSE79150*	DEGs FCH>2.0 FDR<0.05
3	Research study of keratinocyte function in HS [3]	WB HS patients n=18; healthy controls n=18	[@] De-roofed lesional HS skin n=7; HC n=6	Microarray Human HT-12v4 BeadChips (Illumina)	DEGs FCH>1.5 FDR<0.01
4	Serum proteomic signatures in chronic skin disease [4]	Serum moderate-severe AD n=20; Ps n=12; CD n=10; AD+CD n=10, HC n=10		SOMAscan platform, 1129 serum proteins	DEPs FCH>1.5 FDR<0.05

[%] Proteomic serum analysis was conducted during a clinical trial for anti-IL-12/23 (ustekinumab) in the same cohort of moderate to severe HS patients in study 2 (see S1 Fig).
[#] Blok *et al.* compared expression profiles of "inflammatory lesions" (LS) to normal-appearing skin on the upper arm or leg of the same HS patients (NL).
[@] Hotz *et al.* compared the HS skin transcriptome after removal of skin above active HS lesions (LS) ("de-roofing") to skin biopsies from healthy controls. The data from Hotz *et al* was on a different platform and compared LS skin to healthy volunteers, making it difficult to integrate. Results from this analysis are presented in S11 Fig.

References

1 Blok JL, Li K, Brodmerkel C *et al.* Ustekinumab in hidradenitis suppurativa: clinical results and a search for potential biomarkers in serum. *Br J Dermatol* 2016; **174**: 839-46.
2 Blok JL, Li K, Brodmerkel C *et al.* Gene expression profiling of skin and blood in hidradenitis suppurativa. *Br J Dermatol* 2016; **174**: 1392-4.
3 Hotz C, Boniotto M, Guguin A *et al.* Intrinsic Defect in Keratinocyte Function Leads to Inflammation in Hidradenitis Suppurativa. *J Invest Dermatol* 2016; **136**: 1768-80.
4 Wang J, Suarez-Farinas M, Estrada Y *et al.* Identification of unique proteomic signatures in allergic and non-allergic skin disease. *Clin Exp Allergy* 2017; **47**: 1456-67.

Abbreviations

hidradenitis suppurativa (HS), differentially expressed genes (DEGSs), differentially expressed proteins (DEPs), fold change (FCH) false discovery rate (FDR), lesional (LS), non-lesional (NL), Gene Set Variation Analysis (GSVA), psoriasis (PS), atopic dermatitis (AD), contact dermatitis (CD)
