# Supplementary material for: Integrating the skin and blood transcriptomes and serum proteome in hidradenitis suppurativa reveals complement dysregulation and a plasma cell signature
Source: PLoS One. 2018 Sep 28;13(9):e0203672. doi: 10.1371/journal.pone.0203672 (PMC6162087; doi:10.1371/journal.pone.0203672)
Supplement: S3 Table — (PDF) [file pone.0203672.s005.pdf]

S3 Table. Published IPA pathways

HS Skin LS vs NL Transcriptome	HS skin LS vs N transcriptome	HS vs N disease proteomic signature
Blok JL et al. BJD 2016; 174: 1392-4.<br><br>Top ten canonical pathways (IPA)	*Hotz C et al. J Invest Dermatol 2016; 136: 1768-80.*<br>Top enriched pathways relevant for immune response (IPA)	*Blok JL et al. Br J Dermatol 2016; 174: 839-46.*<br>Top ten canonical pathways (IPA)
Granulocyte Adhesion and Diapedesis<br>Agranulocyte adhesion and Diapedesis<br>Atherosclerosis signaling<br>Hepatic fibrosis/ Hepatic stellate cell activation<br>Primary immunodeficiency signaling<br>Communication between innate and adaptive immune cells<br>Dendritic cell maturation<br>Complement system<br>Systemic lupus erythematosus signaling<br>Leucocyte extravasation signaling	Role of IL-17 in psoriasis<br>Interferon signaling<br>Role of PRP in recognition of bacteria and viruses<br>Fcy Receptor-mediated phagocytosis in macrophages and monocytes<br>IL-10 signaling<br>Role of macrophages, fibroblasts, and endothelial cells in rheumatoid arthritis<br>Role of NFAT in regulation of the immune response<br>Complement System<br>Acute phase response signaling<br>Dendritic cell maturation	Natural Killer Cell Signaling<br>Fc Epsilon R1 Signaling<br>Insulin Receptor<br>Ephrin Receptor<br>T Cell Receptor Signaling<br>HGF Signaling<br>Pancreatic Adenocarcinoma Signaling<br>Role of Tissue Factor in Cancer<br>Ovarian Cancer Signaling<br>P13K Signaling in B lymphocytes
