# Supplementary material for: Integrating the skin and blood transcriptomes and serum proteome in hidradenitis suppurativa reveals complement dysregulation and a plasma cell signature
Source: PLoS One. 2018 Sep 28;13(9):e0203672. doi: 10.1371/journal.pone.0203672 (PMC6162087; doi:10.1371/journal.pone.0203672)
Supplement: S8 Table — (PDF) [file pone.0203672.s010.pdf]

S8 Table. Candidate biomarkers across platforms.xlsx

Candidate serum proteins	SERUM PROTEIN (individ) FCH HS v N*	p-value	reference	Serum Proteomics (S6 Table) in somascan?	HS v N FCH HS v N	p-value	HS LS tissue expression	reference	SKIN TRANSCRIPTOME (LS vs NL) (S4 Table) Probe ID	SYMBOL	GENENAME	lgFCH_Skin. LSvsNL	FCH_Skin. LSvsNL	pvals_Skin. LSvsNL	fdrs_Skin. LSvsNL	Status Skin DEG
CRP	5.9	>N IN 56%	Matsuiak, 2015	Y	1.11	0.602			205753_PM_at	CRP	C-reactive protein	0.22	1.16	0.00638	0.0312	0
									37020_PM_at	CRP	C-reactive protein	0.04	1.03	0.517	0.685	0
sIL2R	1.6	=0.001	Matsuiak, 2015	Y (G)	1.29	0.019			204116_PM_at	IL2RG	interleukin 2 receptor subunit gan	1.48	2.79	5.48E-06	0.000195	1
TNF	1.4	=0.006	Matsuiak, 2009	Y	1.06	0.334			ND							
IL-17A	1.5	<0.0001	Matsuiak, 2016	Y	1.03	0.56	YES	Schlapbach, 2011; Lima 2016	216876_PM_s_at	IL17A	interleukin 17A	0.7	1.62	0.0043	0.0232	0
									208402_PM_at	IL17A	interleukin 17A	-0.05	-1.04	0.397	0.58	0
IL6	10.3	=0.001	Jimenez-Gallo, 2017	Y	1.25	0.093			205207_PM_at	IL6	interleukin 6	1.25	2.38	0.000448	0.00439	1
IL32	10.0	<0.05	Thomi, 2017	N			YES	Thomi, 2017	203828_PM_s_at	IL32	interleukin 32	1.37	2.59	2.20E-06	0.000104	1
S100A8	3.4	<0.001	Weiland, 2013	N			YES	Lima, 2016	214370_PM_at	S100A8	S100 calcium binding protein A8	2.97	7.83	8.27E-10	9.27E-07	1
									202917_PM_s_at	S100A8	S100 calcium binding protein A8	2.94	7.65	1.14E-09	1.01E-06	1
S100A9	3.4	<0.001	Weiland, 2013	Y	1.31	0.899	YES	Lima, 2016	203535_PM_at	S100A9	S100 calcium binding protein A9	3.53	11.54	7.68E-09	3.10E-06	1
LCN2	2.1	<0.001	Wolk, 2017	Y	1.15	0.488	YES	Wolk, 2017	212531_PM_at	LCN2	lipocalin 2	2.08	4.24	2.13E-06	0.000102	1
YKL-40, chitinase-like protein 3	1.9	<0.001	Matsuiak, 2015	N					213060_PM_s_at	CHI3L2	chitinase 3 like 2	2.03	4.09	8.69E-09	3.24E-06	1
MMP8	2.8	<0.01	Tsaousi, 2016	Y	1.13	0.187			231688_PM_at	MMP8	matrix metallopeptidase 8	0.2	1.15	0.269	0.452	0
sTNF-RI	2.7	<0.001	Jimenez-Gallo, 2017	Y	1.04	0.517			ND							

*approximate fold change (FCH) calculated from the reference

ND= Not detected on chip
